# Current Landscape and Future Perspectives of Diabetic Retinopathy Therapy: Pharmacological Targets, Precision Laser Technology, and Clinical Evidence

**DOI:** 10.1002/mco2.70849

**Published:** 2026-07-07

**Authors:** Xinying Hu, Zhe Cha, Ao Lu, Wuping Xu, Xiaoqing Zhang, Sheng Miao, Haiwei Xu, Xuedong Xu

**Affiliations:** ^1^ Department of Ophthalmology Affiliated Jiangyin Hospital of Nantong University (Jiangyin People's Hospital) Jiangyin Jiangsu PR China; ^2^ Department of Ophthalmology, Southwest Hospital/Southwest Eye Hospital Third Military Medical University (Amy Medical University), Chongqing Key Laboratory of Visual Injury and Regeneration Chongqing PR China; ^3^ Department of Ophthalmology The First Affiliated Hospital of Chongqing Medical University Chongqing PR China

**Keywords:** diabetic macular edema, diabetic retinopathy, nanocarrier, pharmacological target, subthreshold micropulse laser

## Abstract

Diabetic retinopathy (DR), a major cause of blindness worldwide, poses a substantial and escalating burden on public health. The limitations of current therapeutic modalities highlight the pressing need for continued innovation in therapeutic interventions. This review comprehensively delineated the knowledge architecture, research focal points, and emerging directions in DR therapeutics. Targeted biological agents have recently emerged as principal research directions in DR therapy and hold substantial promise. They are expected to achieve the goals of blocking disease progression more persistently, effectively, and less invasively than nonbiological therapeutics through multitarget synergy and long‐acting sustained‐release formulations. In clinical practice, the paradigm of DR therapy has shifted toward personalized and precision medicine, with optimized subthreshold micropulse laser as a promising adjunctive therapy for enhancing long‐term treatment outcomes. Looking ahead, therapeutic monitoring in DR may evolve beyond conventional morphological grading to incorporate multimodal precision diagnostics, artificial intelligence‐driven broad‐spectrum screening, and biomarker‐based early warning systems. This review concludes with a discussion on prospects and challenges in DR therapeutics, with special emphasis on emerging drug candidates, recent clinical trial findings, and novel insights that may inform the next generation of therapeutic interventions for DR.

## Graphical abstract

Targeted biological agents targeting VEGF, integrin, dopamine D2 receptor, ROCK, and BCL‐XL have entered the clinical trial phase and deserve particular attention.

The treatment paradigm has shifted toward personalized and precision medicine, positioning optimized SML as a promising adjunctive therapy.

Encouraging individualized combination treatment strategies assists in the long‐term clinical management of DR.

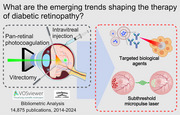

## 1 Introduction

Diabetic retinopathy (DR), characterized by progressive neurodegeneration and microvascular damage in the retina, is a severe complication of diabetes, and has become a leading cause of visual impairment and blindness worldwide [1, 2]. According to epidemiological data in the United States, Australia, Europe, and Asia, the total proportion of diabetes‐related damage in all people with diabetes is approximately 34.6% [3]. Preliminary predictions indicate that the global prevalence of DR will rise significantly over the coming decades, from approximately 103 million individuals in 2020 to 130 million in 2030, with upward of 161 million by 2045 [4]. This trend is driven by a variety of factors, including the rising global prevalence of diabetes, lifestyle changes, increased life expectancy, and global population aging [5]. The resulting burden of DR extends far beyond blindness by impacting family well‐being, labor productivity, the financial sustainability of healthcare, and social equity [3]. DR is a chronic, progressive disease that develops from nonproliferative diabetic retinopathy (NPDR) and may be more serious, progressing to proliferative diabetic retinopathy (PDR) and diabetic macular edema (DME); therefore, it can cause sudden and severe vision loss [2]. NPDR is often asymptomatic and easy to overlook, whereas PDR is the end stage of DR and can lead to severe complications such as vitreous hemorrhage, tractional retinal detachment, and neovascular glaucoma [6], ultimately causing permanent and irreversible vision loss. DME is the most common manifestation of DR, and it represents a major clinical challenge and research focus for ophthalmologists due to its high recurrence rate. Although early diagnosis and intervention can delay disease progression to some extent, vision impairment and the reduced quality of life continue to pose serious threats to patients with DR [7].

**FIGURE 1:**
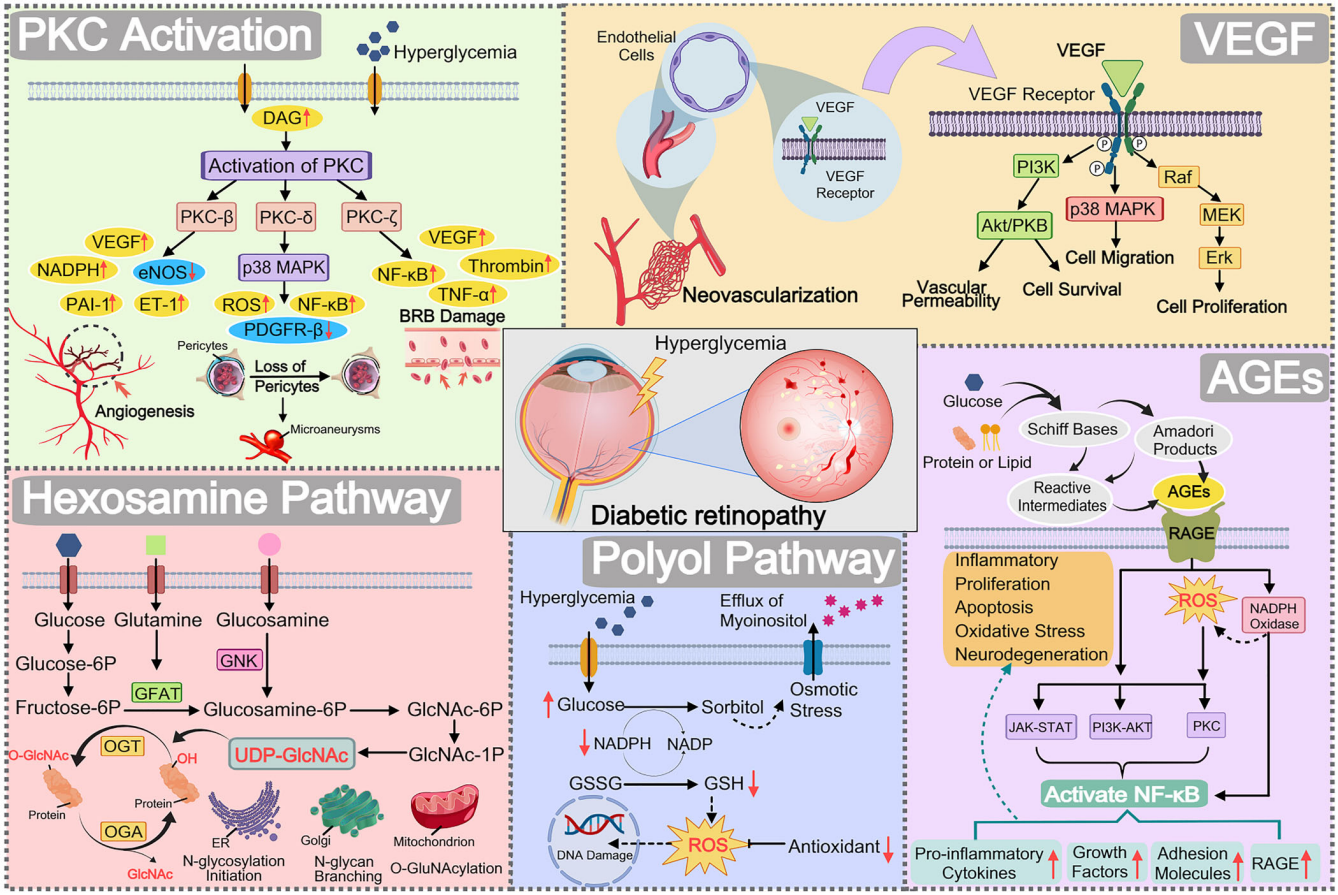
Schematic representation of multiple signaling pathways implicated in the onset and progression of diabetic retinopathy (DR). The pathogenesis of DR involves the interaction of multiple signaling pathways. Hyperglycemia activates the hexosamine pathway, disrupts the polyol pathway, stimulates protein kinase C (PKC) activity, and increases the formation of advanced glycation end products (AGEs). All these factors collectively contribute to the overproduction of reactive oxygen species (ROS). Additionally, retinal cell damage and tissue hypoxia upregulate vascular endothelial growth factor (VEGF), leading to vascular inflammatory destruction, leakage, pericyte loss, and basement membrane degradation. These events ultimately cause blood–retinal barrier (BRB) breakdown and neovascularization. DAG, diacylglycerol; eNOS, endothelial nitric oxide synthase; ER, endoplasmic reticulum; Erk, extracellular signal‐regulated kinase; ET‐1, endothelin‐1; GFAT, glutamine: fructose‐6‐phosphate aminotransferase; GNK, GlcNAc kinase; GSH, glutathione; GSSG, glutathione disulfide; JAK‐STAT, Janus kinase‐signal transducer and activator of transcription; MAPK, mitogen‐activated protein kinase; MEK, mitogen‐activated protein kinase; NF‐κB, nuclear factor kappa‐B; OGA, O‐GlcNAcase; OGT, O‐GlcNAc transferase; PAI‐1, plasminogen activator inhibitor‐1; PDGFR; platelet‐derived growth factor receptor; PI3K, phosphatidylinositol 3‐kinase; PKB, protein kinase B; Raf, rapidly accelerated fibrosarcoma; RAGE, receptor for advanced glycation end products; TNF‐α, tumor necrosis factor‐α; UDP‐GlcNAc, uridine diphospho‐N‐acetylglucosamine. Created with Medpeer.cn.

DR is a disease with multiple factors, pathways, and stages [8]. Accumulating evidence suggests that several key mechanisms contribute to its pathogenesis, including retinal oxidative stress, inflammation, vascular abnormalities, and neurodegeneration [2, 9]. Hyperglycemia activates the hexosamine biosynthetic pathway, disrupts the polyol pathway, stimulates protein kinase C activity, and increases the formation of advanced glycation end products (AGEs) [10, 11, 12]. All of the above are reasons for the overproduction of reactive oxygen species [13]. Then, inflammatory responses induced by oxidative stress increase the levels of various inflammatory cytokines in the eyes, including interleukin‐1β (IL‐1β), interleukin‐6 (IL‐6), and tumor necrosis factor‐α (TNF‐α) [14]. Additionally, retinal cell damage and tissue hypoxia upregulate angiogenic factors, such as antivascular endothelial growth factor (VEGF) and insulin‐like growth factor 1, resulting in vascular inflammatory destruction, leakage, loss of pericytes, and degradation of the basement membrane [12, 15]. These events ultimately cause blood–retinal barrier (BRB) breakdown and neovascularization [16]. Neurodegeneration is another critical pathological feature of DR. Retinal neurons are susceptible to oxidative stress and inflammation, which can trigger degeneration or apoptosis, resulting in visual dysfunction [17]. Figure 1 is a summary of multiple crucial signaling pathways involved in the occurrence and progression of DR.

Over the last five decades, therapeutic interventions for managing sight‐threatening DR have evolved substantially. Presently, clinical interventions can be broadly categorized into three main approaches: systemic therapies, including glycemic control, blood pressure regulation, and lipid‐lowering management; pharmacological agents, such as metabolic regulators, antioxidants, microcirculation enhancers, anti‐VEGF agents, and corticosteroids; and procedural interventions, including retinal laser photocoagulation and vitrectomy [18, 19, 20]. However, these therapeutic approaches possess certain limitations and have not yet achieved fully satisfactory clinical outcomes. The major cause lies in the fact that all of the existing DR therapies focus on passive defense (i.e., eliminating neovascularization and reducing edema), which does not address the fundamental pathogenesis of DR—metabolic abnormalities, inflammation, and microvascular damage. Further, the lack of effective early interventions forces a late‐stage interventional approach. These limitations underscore the urgent need to develop novel or adjunctive therapies for the early prevention and etiological treatment of DR to improve clinical outcomes.

In recent years, various therapeutic approaches on DR have emerged, including targeted agents [21], stem cell [22, 23], gene [24], natural medicines [25], and precision laser therapies [26]. However, the progress in these innovative therapies and challenges in clinical translation have not been adequately characterized. To provide a theoretical foundation and practical insights for developing innovative DR therapies, a comprehensive synthesis evaluating research hotspots and evolving trends across different therapeutic modalities is needed.

**FIGURE 2:**
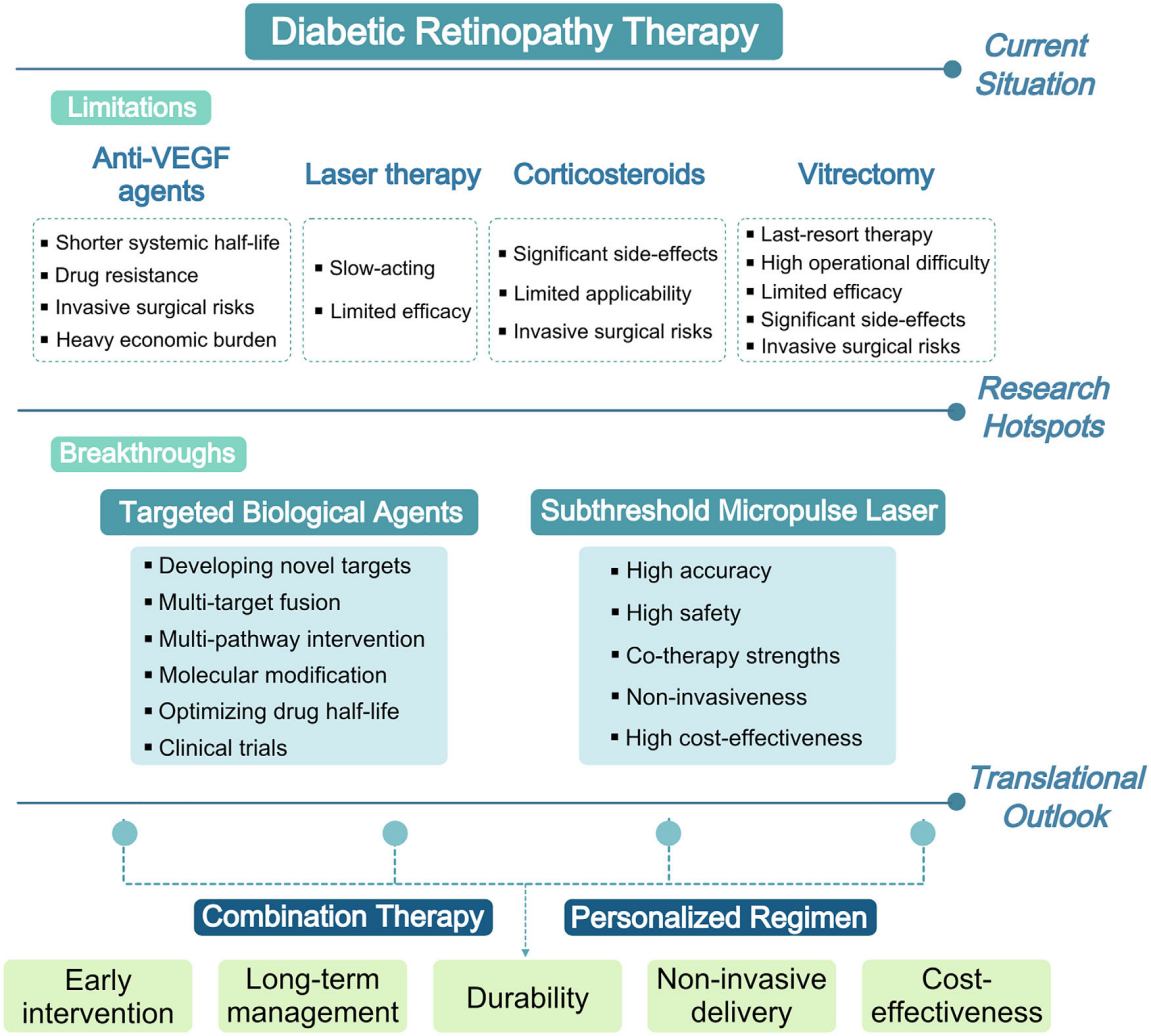
Conceptual framework of diabetic retinopathy (DR) therapy. This diagram synthesizes the current situation, research hotspots, and translational outlook for DR therapy. The existing treatment strategies—including anti‐VEGF agents, laser therapy, corticosteroids, and vitrectomy—present certain clinical limitations (e.g., frequent intravitreal injections, variable patient responses, and inherent invasiveness). In response, two emerging research hotspots have been proposed: targeted biological agents and subthreshold micropulse laser. They hold enormous application prospects (e.g., long‐acting effect, high accuracy, and noninvasiveness) but remain subject to clinical translation challenges. Future perspectives are expected to emphasize long‐term management, noninvasive delivery, and early intervention. VEGF, vascular endothelial growth factor. Created with Medpeer.cn.

Based on our previous bibliometric analysis of 14,875 publications from 2014 to 2024, this review aims to synthesize research across the full spectrum of DR therapy and to establish a practical framework that reflects the current landscape and future perspectives (Figure 2). By presenting the latest overview of DR therapy spanning from pharmacological targets to precision laser technology, and bridging the gap between mechanistic insights and clinical translation, we hope to offer a valuable source for guiding clinical applications and developing innovative therapeutic strategies.

## 2 Pharmacological Therapeutics

Pharmacological therapy is the mainstay of DR management. Most prominently represented by anti‐VEGF agents, targeted biological agents are driving a revolutionary breakthrough in DR pharmacotherapy with strong application prospects. Meanwhile, corticosteroids, leveraging their sustained‐release platforms, offer a long‐acting treatment option. As a complementary approach, traditional Chinese medicine (TCM) has demonstrated distinctive value in early intervention and systemic regulation. Further, nanotechnology‐based drug delivery is expected to reshape the therapeutic paradigm with the deepening of interdisciplinary collaboration. A detailed discussion of this topic is presented in what follows.

### 2.1 Targeted Biological Agents

DR are neurodegenerative and microvascular diseases, the DR‐associated signaling cascades are complicated and might be served as potential drug targets [27, 28]. A series of biological agents have been designed, and have either been approved or are currently in clinical trials, as detailed in Figure 1 and Table 1.

**TABLE 1:** Evidence summary on targeted biological agents for DR.

Typical agents	Therapeutic target	Durability strategy	Administration route	Collective evidence and potential	Translational outlook
Bevacizumab [29] Ranibizumab [30] Aflibercept [31]	VEGF‐A VEGF‐A VEGF‐A, VEGF‐B, PlGF	Initial 4‐week dosing interval, then as needed	IVI IVI IVI	Inhibit VEGF‐mediated neovascularization and rapidly resolve macular edema.	Currently, the first‐line treatment for DME [32]. ^a^Urgent development of local, sustained‐release ocular formulations is critical.
Faricimab [33]	VEGF‐A, Ang‐2	6.1–10.4 weeks injection interval	IVI	Enhances vascular stability, reduces leakage, and prolongs the treatment interval through a dual‐targeting mechanism.	Approved for clinical use by the FDA [34].
Brolucizumab [35]	VEGF‐A	Initial 6‐week dosing interval, followed by 8–12‐week interval	IVI	Its low molecular weight (∼26 kDa) enables delivery of approximately 11‐fold higher molar dose than aflibercept.	Approved for clinical use by the FDA [36].
Risuteganib (ALG‐1001, Luminate) [37]	Integrins: αVβ3, αVβ5, α5β1, αMβ	3 × monthly doses, with 12 weeks’ durability after the final injection	IVI	Inhibits four key RGD integrins, providing better therapeutic effects through multipathway intervention (simultaneously inhibiting inflammation and angiogenesis).	A Phase II clinical trial was completed. (NCT02348918) [38]
THR‐687 [39]	Multiple RGD integrins	Durability for 3 months postsingle injection	IVI	Extends the effects of a single intravitreal injection by up to 3 months, with good tolerance.	A Phase I clinical trial (NCT03666923) demonstrated favorable safety and preliminary efficacy [40], whereas a Phase II trial failed to meet its endpoint.
EGT022 (EG‐Mirotin) [41]	Integrins: αIIbβ3, αvβ3	Administration once daily for 5 consecutive days, with sustained efficacy of 7 weeks	SC	Suppresses abnormal neovascular sprouting and retinal capillary leakage by targeting integrin αvβ3 and αIIbβ3, especially for NPDR.	A Phase II clinical trial was completed. (approval no. 32764) [42]
SF0166 [43]	Integrins: αvβ3	Administration for 4 weeks	Eye drops	Decreases the central retinal thickness in 55% of patients, without pain or injury.	A Phase I/II clinical trial was completed. (NCT02914613) [44]
OTT166 [45]	Integrins: αvβ3, αvβ6, αvβ8	Administration for 4 weeks	Eye drops	Biological activity was detected in 37% of patients.	A Phase Ib clinical trial was completed. (NCT02914613) [45]
Levosulpiride [46]	Dopamine D2 receptor	Administration for 8 weeks	Oral	Improves visual and anatomical outcomes by upregulating angiostatin and downregulating VEGF expression, with no significant adverse effects.	^a^Overcoming poor bioavailability is paramount for clinical success. A Phase II clinical trial was completed. (NCT03161652) [46]
THR‐149 [47]	Plasma kallikrein	Single injection	IVI	Improves vision through its downstream signaling targets, with a favorable safety profile.	A Phase I clinical trial was completed. (NCT03511898) [48] While a Phase II trial failed to meet the endpoint.
Fasudil [49]	ROCK	3 × monthly doses	IVI	Enhances and prolongs the therapeutic effects of anti‐VEGF drugs for DME. Achieves moderate visual improvement for patients with refractory DME.	A pilot randomized clinical trial was completed. (NCT01823081) [49, 50].
Foselutoclax (UBX1325) [51]	BCL‐xL	Single injection (BEHOLD) 8‐week dosing interval (ASPIRE)	IVI	Specifically targets and clears senescent cells in the retina by inhibiting the antiapoptotic protein BCL‐xL, thereby reducing inflammation and vascular leakage. A single intravitreal injection provides a durable, disease‐modifying effect (lasting at least 18–24 weeks), with no significant adverse effects.	^a^Holding unique value in neurodegeneration. Two Phase II clinical trials were completed. (BEHOLD, NCT04857996 [52], ASPIRE, NCT06011798 [53]). The efficacy and durability hold promise for replacing anti‐VEGF drugs in the future.

Abbreviations: Ang‐2, angiopoietin 2; BCL‐xL, B‐cell lymphoma‐extra‐large; DME, diabetic macular edema; FDA, US Food and Drug Administration; IVI, intravitreal injection; NPDR, nonproliferative diabetic retinopathy; PlGF, placental growth factor; RGD, arginyl‐glycyl‐aspartic acid; ROCK: Rho/Rho‐associated protein kinase; SC, subcutaneous injection; VEGF, vascular endothelial growth factor.

^a^Deserves special attention.

#### 2.1.1 Anti‐VEGF

VEGF is the pivotal factor involved in the development of DR, the anti‐VEGF agents including bevacizumab, ranibizumab, and aflibercept have been well‐established and made clinical success, used as first‐line therapy for DME [54]. However, the limitations of current anti‐VEGF treatments are obvious, including the burden of frequent intravitreal injections and variable patient responses, which have driven the development of longer acting and multitarget biologics. Emerging agents have been designed to extend dosing intervals, therapeutic efficacy, and remaining safety considerations.

Bevacizumab (Avastin; Genentech, South San Francisco, California, United States) is a recombinant humanized monoclonal IgG1 antibody that can bind all forms of VEGF‐A [29], and it is the first anti‐VEGF drug to be approved for clinical use. Following structural optimization, ranibizumab (Lucentis; Genentech, South San Francisco, California, United States) is a monoclonal antibody fragment that can bind to and inhibit VEGF‐A isoforms and their biologically active degradation products [30]. On the other hand, aflibercept (Eylea; Regeneron, Tarrytown, New York, United States) is a recombinant fusion protein that has joined the extracellular regions of VEGFR‐1 and VEGFR‐2 with the Fc region of human IgG1 [31]. As a blocker of VEGF‐A, VEGF‐B and placental growth factor, it can reduce abnormal blood vessel formation and leakage by inhibiting these molecules. Several clinical trials have showed the significant efficacy of these three anti‐VEGF agents in improving the vision of patients with DME [55, 56, 57]. However, because of the short half‐life of these drugs, they may not be effective or the disease may develop resistance; therefore, frequent intravitreal injections and adjustments in medication are necessary, and both are expensive and mentally exhausting for the patients [58, 59, 60]. This has prompted ongoing research into longer acting agents that can achieve durable VEGF suppression with fewer administrations.

One such advancement is faricimab (Vabysmo; Roche, Basel, Switzerland), a novel bispecific monoclonal antibody that targets both angiopoietin 2 and VEGF‐A, and promotes vascular stability with extended therapeutic durability. Results from 12 clinical trials have reported a prolonged injection interval with faricimab, with an average of 6.1–10.4 weeks [33]. Notably, over extended study durations (≥ 6 months), patients treated with faricimab consistently maintained visual acuity improvements, with a reduced central macular thickness (CMT), retinal fluid accumulation, and pigment epithelial detachment [33, 61]. Similarly, RC28‐E, another investigational bispecific antibody that targets both VEGF‐A and fibroblast growth factor‐2, is currently undergoing Phase II clinical trials [62]. The development of multitarget biologics represents a promising, and perhaps necessary, future direction for DR therapy.

Brolucizumab (Beovu, Novartis, Basel, Switzerland) is a recently introduced anti‐VEGF agent. It comprises a single‐chain variable fragment with a high binding affinity for VEGF‐A isoforms. Because of its low molecular weight (∼26 kDa), brolucizumab can be delivered at an approximately 11‐fold higher molar dose than aflibercept [35]. A systematic review and meta‐analysis of 1253 patients with DME from three randomized controlled trials (RCTs) showed that brolucizumab was noninferior to aflibercept in terms of functional outcomes and was superior in terms of anatomical parameters [63]. Critically, compared with the monthly administration of aflibercept, the initial 6‐week dosing interval of brolucizumab (extendable to 8–12 weeks) may improve patient adherence, reduce treatment burden, and ultimately lead to better long‐term visual outcomes and lower visual morbidity [64, 65]. However, considering that DME is a chronic, progressive disease that typically requires long‐term treatment and monitoring, the sustained therapeutic efficacy and safety profile of brolucizumab still needs to be fully elucidated in extended follow‐up studies.

#### 2.1.2 Anti‐Integrin

Integrins are a family of adhesion molecules found on cell surfaces. Their name reflects their ability to bridge the extracellular matrix (ECM) with the intracellular actin cytoskeleton [66]. Among them, arginyl‐glycyl‐aspartic acid (RGD)‐binding integrins, such as αvβ3, αvβ5, and α5β1, are considered to be crucial in the pathogenesis of DR [67, 68]. Beyond inflammatory pathways, integrin αvβ3 and αvβ5‐mediated angiogenesis pathways have also been identified [69, 70]. Integrin αvβ3 is expressed on endothelial cells and inflammatory cells in the active proliferation phase [71, 72]. Its activation also maintains the inflammatory response of macrophages [73]. In patients with PDR, the activation of integrin αvβ5 on the retinal fibrovascular membrane promotes the accumulation of fibronectin and alters its radial distribution along sprouting endothelial cells, thereby triggering retinal neovascularization [74, 75]. These findings suggest that anti‐integrin therapy has the potential to become a novel therapeutic intervention to treat DR. Benefiting from its multipathway effects that simultaneously inhibit inflammation and angiogenesis, anti‐integrin therapy may provide more durable efficacy compared to anti‐VEGF therapy [68, 76]. Currently, anti‐integrin therapy remains at the stage of clinical trials, and its safety and long‐term efficacy have not been confirmed.

One integrin inhibitor being investigated is risuteganib (ALG‐1001, Luminate), a synthetic peptide that inhibits four key RGD integrins: αVβ3, αVβ5, α5β1, and αMβ [37]. These integrins are associated with both age‐related macular degeneration and DME [77]. In a Phase II trial, patients with DME received an initial bevacizumab injection, followed by monthly risuteganib injections for 3 months. The results showed that risuteganib was noninferior to monthly bevacizumab, with its effects persisted for up to 12 weeks after the final injection [38]. In terms of safety, a randomized, double‐anonymized controlled study found that risuteganib (1.0 mg) administered twice over 32 weeks produced no serious drug‐related adverse events [78]. To validate these preliminary findings, larger scale trials with a longer follow‐up and more frequent dosing regimens are urgently needed.

Based on the excellent performance of risuteganib, THR‐687, a small molecule integrin antagonist (∼750 Da) was developed, with a low nanomolar affinity for many RGD integrins [79]. The therapeutic potential of THR‐687 was first evaluated in a Phase I, open‐label, multicenter, 3 + 3 dose‐escalation trial [40]. The study reported that a single intravitreal injection was well‐tolerated and led to sustained anatomical and functional improvements, including an 8.3‐letter gain in best corrected visual acuity (BCVA) and a 43.9‐µm reduction in central subfield thickness, which were maintained for at least 3 months [40]. Considering the prolonged effects observed with both risuteganib and THR‐687, integrin inhibitors may reduce the frequency of current anti‐VEGF treatments. Looking forward, combination regimens with both anti‐VEGF and anti‐integrin agents may provide an additional option for DME treatment.

Recent efforts have also focused on modifying the route of administration of integrin inhibitors to improve patient compliance and reduce invasiveness [80]. In 2023, EG‐Mirotin was developed by EyeGene Inc. as a noninvasive alternative for patients with NPDR, which contains the active ingredient EGT022 [42]. EGT022 is a recombinant RGD‐containing disintegrin [81] that specifically binds to integrin αIIbβ3 and αvβ3 [82, 83]. It was reported to suppress abnormal neovascular sprouting by targeting integrin αvβ3 on endothelial cells [83, 84] and bind to integrin αIIbβ3 on the surface of thrombocytes to repair retinal capillary leakage [85, 86]. The latest Phase II clinical trial confirmed the feasibility and safety of EG‐Mirotin administered by subcutaneous injection, with a potential benefit in improving early retinal ischemia and vascular permeability [42]. At the same time, a topically administered integrin αvβ3 antagonist called SF0166 (in the form of eye drops) was evaluated in a Phase I/II clinical trial enrolling 44 patients with DME [44]. Following 28 consecutive days of administration, 38% of patients showed a decrease in central retinal thickness, and 55% showed a decrease on Day 56 [44]. Similarly, OTT166 is another eye‐drop preparation that targets several integrins (αvβ3, αvβ6, and αvβ8), and it has also been found to have biological effects in 37% of patients with DR in a Phase Ib trial [45]. Based on the above, it is believed that noninvasive integrin inhibitors are safe and well‐tolerated, but their clinical efficacy is still relatively low compared with intravitreal agents such as risuteganib and THR‐687. The weak efficacy of topical agents is likely due to poor ocular bioavailability [87]. These results highlight the need for innovative drug delivery, which is summarized in detail in Section 2.4.

#### 2.1.3 Other Pharmacological Targets

Beyond anti‐VEGF therapy, several non‐VEGF therapeutic targets for DR are being explored, with the potential to serve as alternatives or adjuvants for DME treatment. Levosulpiride, a dopamine D2 receptor antagonist, was shown to increase intraocular angiostatin levels and inhibit retinal hyperpermeability [88]. A Phase II randomized clinical trial showed that 8 weeks of oral levosulpiride improved both visual and anatomical outcomes in patients with DME by upregulating angiostatin and downregulating VEGF levels [46]. Moreover, the Rho/Rho‐associated protein kinase (ROCK) signaling pathway, one of the downstream cascades of transforming growth factor‐β, is also involved in the progression of DR [89]. A prospective interventional case series study of patients refractory to conventional therapies reported that intravitreal injection of fasudil, a ROCK inhibitor, was safe and moderately effective in improving visual function, but did not consistently show significant anatomical improvements [49, 50]. In addition to the VEGF pathway, the kallikrein–kinin system also promotes DR progression. In particular, bradykinin promotes inflammation, vasodilation, and vascular leakage through its downstream signaling [90]. THR‐149 is a novel, potent inhibitor of the bradykinin‐releasing protease plasma kallikrein. A Phase I study showed the excellent safety and tolerability of intravitreal THR‐149, along with preliminary efficacy in DR as revealed by improved BCVA [48]. In recent years, a novel therapeutic direction targeting senescent cells in the diabetic retina, senolytic therapy, has been investigated [51]. Two Phase II prospective, multicenter, randomized studies evaluating senolytic therapy were completed with exceptional results [52]. In patients with advanced DME who were unresponsive to anti‐VEGF agents, senolytic therapy, exemplified by the B‐cell lymphoma‐extra‐large (BCL‐xL) inhibitor foselutoclax (UBX1325), specifically targeted and cleared senescent cells in the retina by inhibiting the antiapoptotic protein BCL‐xL, thereby reducing inflammation and vascular leakage [51, 52, 53]. Notably, a single intravitreal injection of foselutoclax provided a durable, disease‐modifying effect lasting at least 18–24 weeks without significant adverse effects, positioning foselutoclax as a potential alternative to anti‐VEGF therapy for DME as a first‐line treatment [52]. These studies on therapeutic targets offer new insights for personalized treatment of DR; however, further clinical validation is needed before these become practical applications.

#### 2.1.4 Preclinical and Clinical Studies

In recent years, significant progress has been made in preclinical and clinical studies evaluating biological targeted agents that address different pathological aspects of DR. Preclinical animal studies are irreplaceable in mechanistic explorations and preliminary screening, yet their translational predictive value is inherently limited. To provide a clear understanding of the evolving therapeutic landscape, we highlight here DR‐targeted biological agents that have been approved for clinical use or are currently undergoing clinical trials.

As previously stated, extensive clinical trial validation has established anti‐VEGF agents as the undisputed first‐line treatment for DME, despite their insufficient durability [55, 56, 57]. On this basis, the next‐generation anti‐VEGF agents, exemplified by faricimab and brolucizumab, have successfully overcome the limitations of traditional anti‐VEGF therapies with their notably favorable durability profiles [33, 35]. In contrast, the development of integrin inhibitors and other biological targeted agents remains at the clinical trial stage. Although these agents have considerable application potential, they also present non‐negligible translational challenges, warranting close attention to their progress. Table 1 comprehensively compares these targeted biological agents, including their pharmacological targets, clinical translation potential, and current clinical trial phase.

### 2.2 Steroids

Steroids were the first‐generation intravitreal injection drugs used to treat DME. Due to its high efficacy and low cost, intravitreal steroid injection is now an established second‐line therapy for DME [91]. However, prolonged use of steroids increases the risk of adverse ocular outcomes, including glaucoma and cataract formation, which cause the further distress and economic loss on patients [92]. When making clinical decisions, physicians must carefully weigh the significant antiedema efficacy against well‐established steroid‐related side effects. At present, three fluorinated corticosteroids—triamcinolone acetonide (TA), dexamethasone (DEX), and fluocinolone acetonide (FA)—and their intravitreal implant formulations have been used in the clinical treatment of DME [93].

As illustrated in Figure 1, chronic inflammation plays a central role in the pathogenesis of DR and steroid therapy act as a multitarget anti‐inflammatory approach. Specifically, steroids inhibit the production of inflammatory factors, and stabilize the membranes and tight junctions of vascular endothelial cells and retinal pigment epithelial (RPE) cells to repair the BRB and promoting the absorption of macular effusion [94, 95]. Moreover, intravitreal steroid injection can also downregulate VEGF expression, inhibit leukocyte migration and adhesion, and exert antifibrotic effects; thus, it can also be a valuable adjuvant therapy for PDR and served as a preoperative step to for optimizing vitrectomy conditions [96, 97].

TA, DEX, and FA are the representative fluorinated synthetic corticosteroids lacking mineralocorticoid activity used clinically to treat DME [93]. They differ in their glucocorticoid receptor binding affinities, duration of action, and lipophilicity, which may partially explain their relative potencies [98]. Intravitreal TA injection was first used in 2001 to treat recalcitrant diabetic maculopathy. Since then, multiple studies have demonstrated that intravitreal TA injection improves vision and reduces macular thickness in eyes with refractory DME [99, 100]. However, although intravitreal TA injection has exhibited remarkable efficacy in treating DME, is routinely used in clinical practice, and is strongly supported by evidence‐based medicine and authoritative clinical guidelines, it has not yet received approval from the US Food and Drug Administration (FDA) [101]. This may be due to TA suspension issues, as the suspension tends to have an uneven intraocular drug distribution and unstable drug concentration, which can increase the risk of cataracts and glaucoma. Further, according to Beer et al., the average elimination half‐life after a single intravitreal injection of TA was shown to be 18.6 days, and in vitrectomized patients, the half‐life was even shorter at only 3.2 days, indicating a need for repeated TA injections [102]. In comparison, continuously released low‐dose steroids may yield better therapeutic effects with fewer cataract or glaucoma complications, which has spurred the development of DEX and FA sustained delivery devices.

Intravitreal implants of both DEX (Ozurdex; Allergan, Irvine, California, United States) and FA (Iluvien; Alimera Sciences, Alpharetta, Georgia, United States) have received FDA approval for the management of DME, representing a significant advancement in the dosage form and technology of steroid therapy [103]. The DEX implant is a biodegradable solid polymer implant that can be delivered to the posterior segment of the eye through a 20‐gauge transscleral incision, where it slowly releases DEX [104]. In 2007, Kuppermann et al. first evaluated the efficacy and safety of DEX implants in patients with macular edema in a randomized, multicenter, dose‐ranging controlled clinical trial [105]. Their results indicated that treatment with a single DEX implant significantly improved BCVA in patients with persistent DME, with the therapeutic effect peaking at 90 days and extending up to 180 days when well‐tolerated in patients. In addition, visual acuity and anatomic responses tended to be more pronounced with the 0.7‐mg dose of the DEX implant than with the 0.35‐mg dose [105, 106]. As with all intraocular steroids, the side effects of the DEX implant are critical considerations in clinical decision‐making. Compared to TA and FA, DEX has the lowest lipophilicity and therefore the least accumulation in the trabecular meshwork and lens; as a result, its use may reduce the relative risk of elevated intraocular pressure (IOP) and cataract progression [107].

The FA implant is a nonbiodegradable microtubule implant that is inserted into the vitreous cavity via a 25‐gauge needle [108]. It continuously and stably releases FA at an extremely low rate (0.2 µg per day) for up to 36 months [109], representing the pinnacle of ultra‐long‐acting sustained‐release technology. Two pooled, authoritative trials of DME patients showed that those previously treated with laser photocoagulation derived greater benefits from a FA intravitreal implant than from a sham injection, with visual acuity improved by more than 15 letters at both 24 and 36 months [109, 110]. Unlike the DEX implant, the FA implant is nonbiodegradable and remains permanently in the eye, which likely increases the incidence of cataracts (82%), glaucoma (37%), and implant‐related complications [111]. Therefore, the FA implant may be more suitable for treating patients with chronic DME resistant to previous treatments (laser and anti‐VEGF therapy), or with aphakia or intraocular lenses.

### 2.3 TCM

**FIGURE 3:**
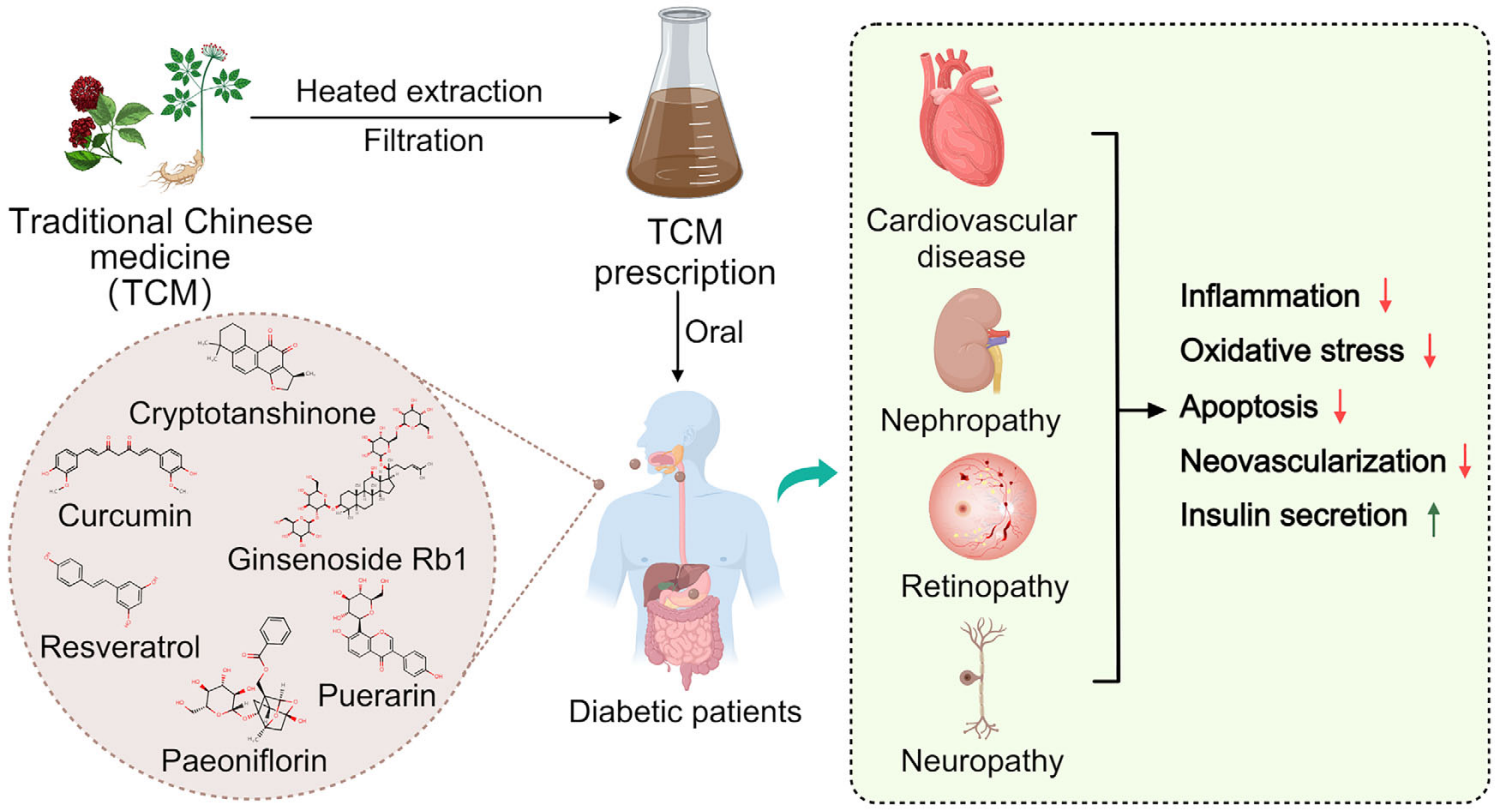
Holistic regulatory effects of oral traditional Chinese medicine (TCM) for diabetic patients. TCM is characterized by multitarget pharmacological properties and noninvasive administration routes. Through the modulation of inflammation, alleviation of oxidative stress, inhibition of apoptosis, and suppression of neovascularization, it exerts holistic regulatory effects and holds promise for improving systemic complications of diabetes. Created with Medpeer.cn.

With ongoing modernization and advances in research methodologies [112, 113, 114], TCM is a promising routine adjunctive therapy for preventing and managing DR. In contrast to modern biological agents, it is characterized by multicomponent therapeutics with multitarget pharmacological mechanisms [115, 116] that produce holistic regulatory effects through multiple pathways (Figure 3). In clinical practice, TCM treatments support personalization and optimization via syndrome differentiation and tailored herbal formulations [117]. Further, TCM offers advantages such as noninvasive administration routes (e.g., oral and topical applications) and high cost‐effectiveness, rendering it suitable for use across all stages of DR, particularly early prevention and stable‐phase maintenance. Numerous studies have demonstrated that TCM can modulate inflammatory responses [118, 119], alleviate oxidative stress [120, 121], inhibit apoptosis [122, 123], and suppress neovascularization [124], thereby potentially mitigating DR progression. Due to the complexity of TCM formulations and challenges in identifying active components and mechanisms, this review focuses on monomeric TCM compounds with well‐defined molecular structures that support mechanistic investigations. Table S1 summarizes therapeutic pathways and targets, the clinical potential, and translational prospects of representative TCM monomers in DR research.

### 2.4 Nanotechnology‐Based Drug Delivery

**FIGURE 4:**
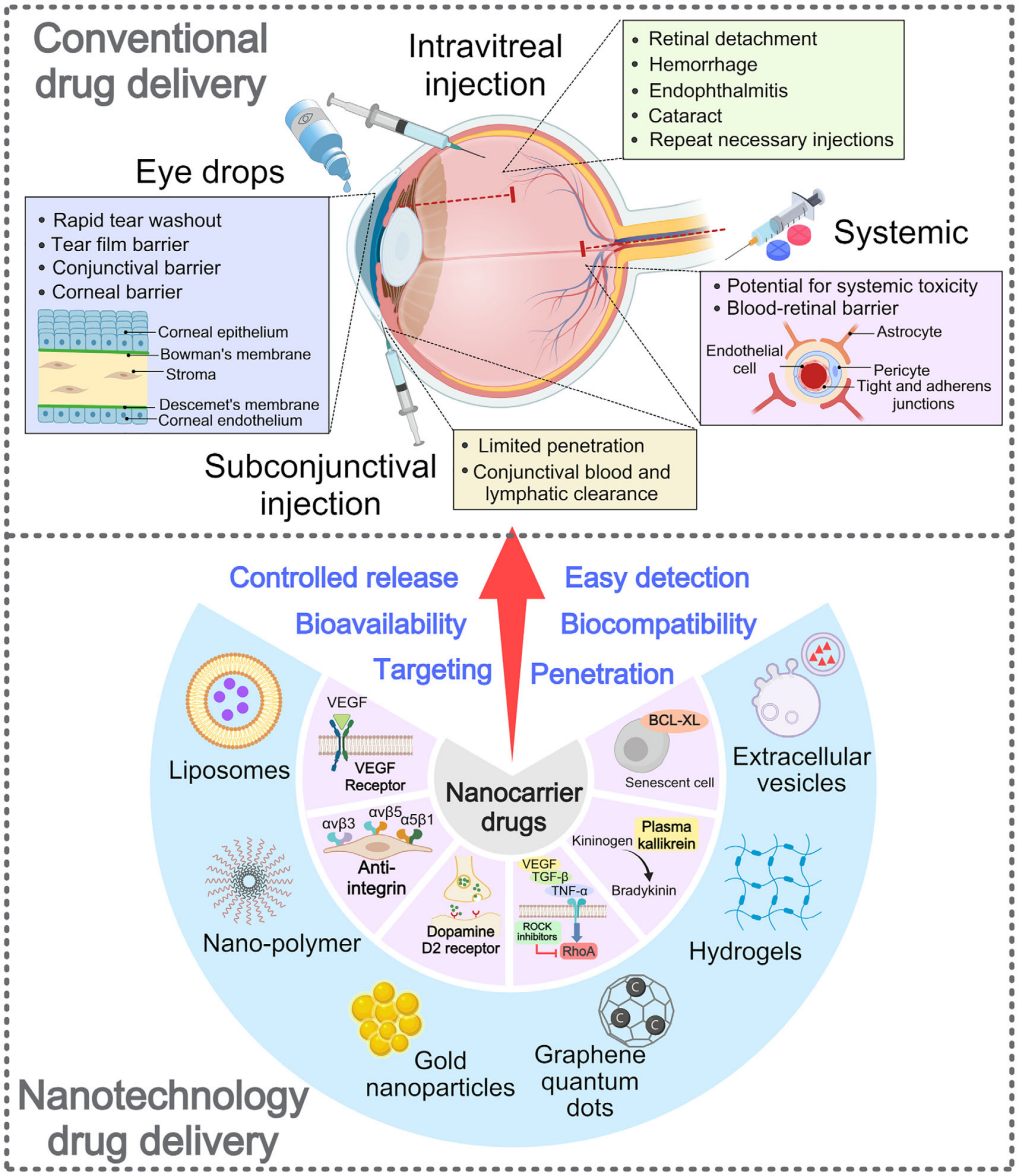
Drug deliver innovation for ocular posterior segment diseases: nanocarriers and targeting technology. Conventional drug delivery systems face clinical limitations, primarily reflected in poor ocular bioavailability and inadequate penetration of ocular biological barriers. Nanocarriers, with their targeting, controlled‐release, and tissue‐penetrating advantages, are expected to reshape the therapeutic paradigm. Their integration with biological targeted agents represents a key priority for future research. ROCK, Rho‐associated protein kinase; TGF‐β, transforming growth factor‐β; TNF‐α, tumor necrosis factor‐α; VEGF, vascular endothelial growth factor. Created with Medpeer.cn.

A major challenge of current pharmacological therapeutics for DR is their limited ocular bioavailability, which arises primarily from the eye's complex anatomical structure and physiological barriers such as the BRB and corneal epithelium [125, 126]. With growing interdisciplinary collaboration, nanotechnology‐based drug delivery has emerged as a transformative platform facilitating targeted delivery, controlled release, and tissue penetration [127, 128]. In recent years, diverse nanocarrier systems have been actively explored for DR to enhance drug bioavailability and minimize systemic side effects [129, 130] (Figure 4). Their respective application prospects and representative preclinical evidence merit systematic summary and in‐depth exploration.

Lipid nanoparticles (LNPs) are regarded as pioneers in nanomedicine due to their high biocompatibility, favorable safety profile, and appropriate particle size (typically 50–1000 nm) [131]. Amadio et al. used a diabetic rat model to evaluate solid LNPs and liposome‐encapsulated siRNA targeting human antigen R (HuR). The results confirmed that the nanocarriers successfully crossed the BRB to deliver the therapeutic microRNAs to the site of action and reduce the expression of HuR and VEGF, alleviating the histological changes caused by DR [132]. In contrast, the advantages of polymer nanomaterials include excellent biocompatibility, low mucosal toxicity, and precise control over drug release kinetics, thereby improving therapeutic stability and prolonging drug efficacy [133]. Liu et al. developed polymeric microspheres encapsulating bevacizumab that enable sustained release for 50 days, demonstrating potential for long‐term intraocular treatment [134]. Pandit et al. further reported that, following subconjunctival administration, chitosan‐coated nanoparticles were able to efficiently deliver bevacizumab to the posterior segment of the eye [135], highlighting their potential in noninvasive ocular drug delivery. Metal‐based nanomaterials are composed of pure metals, such as gold, silver and iron, or metal oxides. Owing to their distinctive physicochemical characteristics (e.g., optical and magnetic properties), these materials exert anti‐inflammatory and antiangiogenic effects through several mechanisms, including oxidative stress induction, cell membrane disruption, and controlled metal ion release [136]. For example, gold nanoparticles were shown to inhibit the expression of VEGF and IL‐1β by blocking the Src kinase pathway, suggesting therapeutic potential in proliferative vitreoretinopathy [137].

Carbon‐based nanomaterials, including graphene derivatives, carbon nanotubes, and carbon dots, are nanoscale materials consisting entirely of carbon atoms. Recently, these materials have been widely applied in biosensing, bioimaging, and drug delivery due to their photostability, low toxicity, cost‐effectiveness, and ease of synthesis [138]. Zhao et al. demonstrated that graphene quantum dots effectively inhibited the proliferation and migration of human umbilical vein endothelial cells and significantly reduce nonperfused and neovascularized areas in a mouse model of oxygen‐induced retinopathy, suggesting their utility in DR therapy for microvascular regulation [139]. Hydrogels, which mimic the ECM, are three‐dimensional polymer networks formed through physical or covalent crosslinking. Hydrophilic polymers can absorb over 90% of their weight in water within their porous, mesh‐like structures, which can also encapsulate hydrophilic drugs and even living cells [140]. With their sustained drug release, high bioavailability, and tunable mechanical properties, hydrogels have considerable advantages as ophthalmic drug delivery agents [141]. Extracellular vesicles (EVs) are nanosized lipid bilayer vesicles, typically 30–150 nm in diameter, which are naturally secreted by most cell types. Compared to synthetic nanocarriers, which may pose immunogenicity and cytotoxicity risks, EVs provide a biologically derived, endogenous delivery platform with very high biocompatibility and minimal immune rejection [142]. Growing evidence indicates that EVs directly modulate multiple DR‐related pathophysiological processes by influencing proliferation‐associated genes, antioxidant response elements, inflammatory mediators, and VEGF expression, thereby attenuating retinal inflammation, neovascularization, microvascular damage, and vascular leakage [143, 144].

## 3 Laser Technology

Panretinal photocoagulation (PRP) has long been one of the main treatment methods for DR. There are some inherent risks and problems in applying it to people. Since the 2000s, with the advent of anti‐VEGF therapy as the new first‐line treatment for DR, PRP has shifted from a primary treatment modality to an important adjunctive therapy. To overcome the limitations of traditional PRP, modern retinal laser technology has been developed to improve the accuracy, safety, and personalization of laser technology.

### 3.1 PRP

In the 1980s, a first‐class study showed that PRP could reduce the number of severe vision‐loss cases due to PDR by over half within 2 years, and thus transform the management of DR [145, 146]. Since then, PRP has become the sole gold standard for treating PDR and severe NPDR. The therapeutic effect of retinal laser photocoagulation is caused by the absorption of light in ocular pigments, which are mainly located in the RPE [147]. Pigments absorb the energy of the laser and generate heat, leading to a localized temperature rise which induces damage to the target retinal cells [148]. In accordance with the main idea of “sacrificing the local for the benefit of the whole,” PRP can reduce the total oxygen consumption and hypoxia in the retina by damaging high‐oxygen‐demand photoreceptor cells in areas with insufficient blood supply [149]. As a result, more oxygen will be supplied to the remaining healthy parts of the retina; therefore, there will be a reduction in pathological VEGF production and consequently, a decrease in retinal vascular permeability and edema [149].

Guided by the results of large multicenter clinical trials, including the Early Treatment Diabetic Retinopathy Study (ETDRS) [145], the standard protocol for traditional PRP has been established as follows: pulse duration of 100–200 ms, laser spot diameter of 100–500 µm, power of 100–750 mW, and 1000–2000 moderate‐intensity burns on the peripheral retina. The complete PRP procedure can be divided into two to four sessions in order to minimize side effects and patient discomfort when using a single‐point laser system [146, 150]. However, despite the benefits in patients with DR, PRP also introduces inevitable complications, such as peripheral vision loss, visual‐field narrowing, reduced contrast sensitivity, and limited driving ability [151]. More serious complications include choroidal detachment, elevated IOP, and cystoid macular edema exacerbation [152].

### 3.2 Modern Retinal Laser Technologies

Pattern scanning laser photocoagulation (PASCAL), navigated laser photocoagulation (NAVILAS), and subthreshold micropulse laser (SML) represent the innovations in laser technology. PASCAL and NAVILAS offer significant improvements over traditional PRP in precision, safety, and patient comfort, while their underlying biological effects remain inherently destructive. PASCAL can select various laser scanning patterns—such as arc, grid, and circular—based on the retinal anatomical structure and clinical indications of patients. By optimizing the exposure duration and spot space, this laser treatment maintains the excellent therapeutic effects of traditional PRP without retinal tissue damage and adverse effects [19]. Unlike traditional laser photocoagulation, which relies on sequential single‐point application, PASCAL utilizes shorter pulses of 10–30 ms to rapidly act on multiple points (4–56 burns) within a defined pattern [153]. A RCT was conducted to compare the efficacy of traditional PRP with PASCAL in the treatment of PDR or severe NPDR, which found that PASCAL produced lesion regression similar to that in conventional PRP but with fewer collateral tissue injuries [154]. Many histological studies have also confirmed that burns with a long pulse duration (> 100 ms) damage the RPE, photoreceptor cells, inner nuclear layer, ganglion layer, and nerve fiber layer simultaneously, while burns with a short pulse duration are less harmful to the inner retina [155]. Additionally, PASCAL can be used for DME. Its circular or arc‐shaped pattern template can safely and accurately locate the foveal exclusion zone, ensuring that the distance between the laser burn spot and the fovea is not less than a preset value.

NAVILAS is an image‐guided, eye‐tracking laser delivery system in which previous fundus examination results (such as fundus fluorescein angiography, fundus autofluorescence, and color fundus images) are digitally overlayed and matched with real‐time fundus views to perform specific and targeted retinal laser photocoagulation in a predetermined and highly precise manner [156]. With its preplanning of the laser area before laser photocoagulation and computer navigation systems, NAVILAS is highly time efficient, enables precise targeting, and offers high safety, thus supporting the development of personalized precision treatment [157]. In clinical practice, PASCAL and NAVILAS are commonly applied as advanced techniques of PRP to treat PDR or severe NPDR. However, the biological effects (destruction or occlusion) of these two laser therapies have not been fundamentally transformed. For DME therapy, SML may represent a more favorable physical therapeutic option.

### 3.3 SML Therapy

The development of SML therapy for DME represents a conceptual revolution in laser technology, with a transition from high‐energy destruction to low‐energy biological regulation. In the mid‐to‐late 2000s, a small‐scale clinical study confirmed for the first time that micropulse laser with subthreshold energy safely alleviated macular edema, improved or stabilized vision, and thus effectively treated DME without leaving any visible laser scars [158]. As shown in Figure S1, unlike traditional retinal laser photocoagulation with its continuous exposure for a set duration, SML therapy delivers short, high‐frequency micropulses that confine heat to the RPE and minimize conduction to surrounding tissue, effectively preserving photoreceptor integrity while providing therapeutic effects [159]. SML also induces bioregulatory effects in RPE cells by promoting the expression of heat shock protein 70. This facilitates damaged RPE protein refolding and blocks inflammatory pathways, thereby accelerating the absorption of macular effusion [160].

Currently, the most common wavelengths used in SML therapy are 577 and 810 nm [161, 162]. An 810‐nm laser, with deeper penetration, targets the RPE and the choroid, whereas the 577‐nm wavelength is mainly absorbed by the RPE. Both have been applied to the treatment of DME [163]. Given that lutein exhibits minimal absorption of 577‐nm yellow light, 577‐nm SML therapy is safer near the fovea, making it the preferred option for center‐involving DME [164, 165]. In 2015, Vujosevic et al. also proposed that 577‐nm SML therapy was superior to 810‐nm SML therapy for DME efficacy [166]. Our previous meta‐analysis assessed the efficacy and safety of SML for the treatment of DME across different parameter settings [167]. Typically, the spot size in the macular region ranges from 100 to 200 µm, with 50 µm near the fovea [168]. A duty cycle of 5% has been confirmed to be safe, whereas duty cycles of 10%–15% have been associated with a risk of retinal thermal damage, although the damage is generally temporary [169, 170]. For the posterior pole and peripheral retina, exposure times of 200–400 ms are generally used, while shorter durations of 50–100 ms are preferred for the macular region. The laser power is titrated for individuals, typically by calibrating the energy on the retina adjacent to the vascular arcade to define the threshold power. Starting at 400 mW, the laser power is gradually increased until a barely visible gray‐white burn (Level I spot) is observed; this is defined as the threshold power. The treatment power is then set to 40%–50% of this threshold to ensure subthreshold delivery [171].

SML is extremely safe, so there are no risks of visual‐field defects, thinning of the retina, and accidental damage to the macula, as with traditional lasers. Thus, it can be used repeatedly in the treatment. SML is not the primary treatment for all types of DR but rather a precise tool specific to certain situations, with safety as its core advantage. According to clinical studies, SML can be considered as an initial treatment option for noncentral DME [172]. For central DME, anti‐VEGF therapy is still the first‐line standard treatment, but SML can be used as an adjuvant or in combination with anti‐VEGF therapy to enhance its therapeutic effect and extend the retreatment interval in some cases, such as when anti‐VEGF therapy is not effective, edema recurs, or the frequency of injections needs to be reduced. However, SML is still in the exploratory stage of the treatment for PDR and cannot replace traditional PRP.

## 4 Clinical Evidence Synthesis and Comparative Efficacy

Although anti‐VEGF agents have become the first‐line treatment for DME, issues such as poor patient adherence due to frequent injections, limited real‐world efficacy, and drug resistance in some patients have driven the development of new‐generation, long‐acting, multitarget anti‐VEGF agents (e.g., faricimab and brolucizumab) as an important direction. Meanwhile, corticosteroids—particularly the DEX intravitreal implant—serve as a powerful weapon against refractory, inflammatory DME owing to their strong anti‐inflammatory effects. For persistent DME, combination therapy of anti‐VEGF agents and steroids has shown additional anatomical benefits, but universally recognized guidelines are still lacking. Furthermore, SML therapy has emerged as an important adjunct to anti‐VEGF treatment for DME, valued not for superior single‐agent potency but for its high safety profile, minimal tissue damage, and suitability for long‐term disease management. Nevertheless, the optimal SML and anti‐VEGF combination protocol remains to be defined. The following sections will elaborate on the clinical evidence, comparative efficacy, and prospects of anti‐VEGF agents, steroids and SML in the management of DME.

### 4.1 Intravitreal Anti‐VEGF Agents: The Cornerstone of First‐Line DME Treatment

Since their introduction in the late 2000s, anti‐VEGF agents have shown good results and are now the first‐line treatment for DME [59]. Currently, the anti‐VEGF drugs commonly used for the treatment of DME in clinical practice are bevacizumab, ranibizumab, aflibercept, faricimab, and brolucizumab [103]. Among them, bevacizumab, ranibizumab, and aflibercept are the most widely used drugs around the world. In 2017, a large‐scale RCT was carried out to compare the efficacy of the three anti‐VEGF agents—aflibercept (2.0 mg), bevacizumab (1.25 mg), and ranibizumab (0.3 mg)—in the treatment of DME [173]. A total of 650 participants received intravitreal injections every 4 weeks over a 2‐year period, and at this time, both the progression and regression of retinopathy were monitored. At the 1‐year follow‐up, for patients with NPDR and DME, bevacizumab demonstrated a lower improvement rate (22.1%) than that of aflibercept (31.2%) and ranibizumab (37.7%). However, by the 2‐year follow‐up, the differences in efficacy among the three agents were no longer statistically significant. In contrast, among patients with baseline PDR, aflibercept yielded a relatively large improvement at both 1 and 2 years (75.9%) than both bevacizumab and ranibizumab [173].

However, due to poor patient adherence of the required high frequency of intravitreal injections, the outcomes of anti‐VEGF therapy in real‐world settings, particularly long‐term visual outcomes [174, 175], are often inferior to those observed in clinical trials [176, 177]. In clinical practice, the efficacy of traditional VEGF drugs typically lasts for about a month, with monthly intravitreal injections required to maintain therapeutic effects [178], which can cause increasingly severe surgical trauma and financial burdens. Following a loading period (3–6 consecutive injections), some patients can extend the treatment interval [179, 180]; however, due to drug resistance, approximately 30%–60% of patients still fail to achieve the goal of no fluid accumulation [181, 182]. To obtain the best results from anti‐VEGF therapy when treating DME, timely assessments of disease progression are essential (initiating treatment when CMT > 250 µm [183]), along with adherence to individualized treatment regimens, including pro re nata (as‐needed injections based on disease activity at regular visits) and treat‐and‐extend (injection interval based on disease activity at the last visit) [171, 184, 185].

Encouragingly, new‐generation anti‐VEGF agents, including faricimab and brolucizumab, launched in some countries have rapidly gained global attention due to their ability to extend treatment intervals. Due to its dual‐target mechanism of action, faricimab can extend the treatment interval to 6.1–10.4 weeks or even longer, according to clinical trial results [33]. Brolucizumab, currently the smallest recombinant humanized single‐chain antibody fragment, can be delivered at an approximately 11‐fold higher molar dose than aflibercept [35]. Compared with traditional anti‐VEGF drugs, brolucizumab has demonstrated noninferior efficacy in vision improvement and macular edema reduction and can extend the treatment interval to 8–12 weeks [64, 65]. The emergence of long‐acting, multitarget anti‐VEGF agents represents a new trend in DR therapy development and may become an option for patients who need to reduce the injection frequency.

### 4.2 Steroids as a Powerful Weapon Against Refractory, Inflammatory DME

Anti‐VEGF therapy and corticosteroids can both improve the vision of patients with DME. From 2000 to 2020, many RCTs have been carried out to compare the effect and safety of intravitreal steroids with that of anti‐VEGF drugs for DME [186]. Due to different designs of the study, various groups of patients, various treatments and different indicators of the outcome, the results of these RCTs were inconsistent. In 2024, Kumar et al. carried out a meta‐analysis of nine clinical trials involving 877 participants, and according to the results, steroids can accelerate the improvement of BCVA and CMT but at the same time increase the risk of IOP and glaucoma; whereas anti‐VEGF agents provided greater long‐term benefits [187]. This finding further confirmed the first‐line status of anti‐VEGF therapy. Recently, long‐acting and sustained‐release forms of steroids, such as DEX implants, have been applied in clinical practice to solve the problems of frequent injections, short duration of action and low patient adherence [188]. Moghib et al. compared the safety and efficacy of intravitreal aflibercept injection versus the DEX implant in the treatment of macular edema [189]. The DEX implant was superior to aflibercept in improving BCVA at 3 months and maintained higher efficacy in reducing CMT at 12 months post‐treatment. However, the DEX implant carried a higher risk of increased IOP at 3 and 6 months, although this risk was manageable [189]. Intravitreal anti‐VEGF injection and steroid injection, particularly long‐acting steroid formulations, are therefore effective methods for managing DME, with each having distinct advantages. Personalized treatment strategies should consider the nature of the DME (e.g., VEGF‐driven, inflammatory, or mixed), severity, duration of diabetes, complications, and individual preferences.

DME treatment must essentially be a multitarget intervention aimed at the different pathways in the complex pathogenesis of DME. Research has shown that over 40% of patients still have persistent DME despite receiving adequate anti‐VEGF therapy [190]. Whether a combination of steroid and anti‐VEGF therapy can achieve additive effects in refractory DME warrants further investigation. A meta‐analysis of eight clinical trials conducted in 2025 revealed that, the combination of DEX implants and anti‐VEGF agents was anatomically superior to anti‐VEGF monotherapy in reducing CMT in the management of DME [191]. Grad et al. also confirmed that this combination treatment yielded more significant BCVA improvements after 4–6 months while maintaining a favorable safety profile in terms of IOP‐related complications [192]. This highlights the potential value of the DEX implant as an adjunct to anti‐VEGF monotherapy. However, no universally recognized guidelines for steroid combination therapy have yet been developed [193].

### 4.3 SML as an Adjuvant or Combination Therapy to Optimize Anti‐VEGF Efficacy

SML is an important supplement to traditional laser photocoagulation and anti‐VEGF therapy. Although the efficacy is relatively limited, its advantages include high safety, minimal side effects, and suitability for long‐term management. In clinical practice, SML therapy can be performed as a simple, minimally invasive outpatient procedure that is generally well‐tolerated [194]. Compared with intravitreal injection, SML is often more acceptable to patients due to its significant economic advantages and superior comfort [195, 196]. Currently, SML therapy is frequently used as an adjuvant treatment with VEGF therapy for patients with DME, particularly those with refractory DME [197]. Several randomized clinical trials have evaluated the efficacy and safety of SML combination therapy for treating DME compared to anti‐VEGF monotherapy. The results consistently demonstrated that SML combination therapy yielded anatomical and visual outcomes comparable to those of anti‐VEGF monotherapy but with the recurrence rate and number of intravitreal injections significantly reduced [194, 198]. Clearly, SML adjuvant or combination therapy can maintain or even exceed the efficacy of anti‐VEGF monotherapy while prolonging treatment intervals and reducing recurrence risks [197].

However, the optimal combination of SML and anti‐VEGF agents is yet to be determined. Table 2 summarizes the current SML and anti‐VEGF therapy combination regimens, including SML parameters, to provide guidance for clinical implementation. Recent clinical studies on combination regimens of SML with anti‐VEGF therapy have typically initiated SML therapy after completing an anti‐VEGF loading phase. Following the loading period (3–6 consecutive injections), SML therapy is commonly administered monthly or guided by the CMT. Although SML therapy combined with anti‐VEGF therapy has become increasingly integrated into clinical workflows, designing cost‐effective and individualized regimens requires a multifactorial clinical assessment. Prognosis, drug tolerance, adherence, and financial capacity should all be considered in therapeutic decision‐making.

**TABLE 2:** Regimens and parameters of SML combined with anti‐VEGF therapy.

Study (year)	Anti‐VEGF agent	Laser wavelength	Spot size Duty cycle Exposure time	Exposure dosage and timing
Moisseiev et al., 2018 [199]	Ranibizumab	577 nm	200 µm 5% 200 ms	3 × monthly IVI 0.3 mg/0.05 mL +SML 2 months postloading dose
Abouhussein et al., 2020 [198]	Aflibercept	577 nm	200 µm 5% 200 ms	3 × monthly IVI 2 mg/0.05 mL + SML 1 month later postloading dose
Khattab et al., 2019 [200]	Aflibercept	577 nm	200 µm 5% 200 ms	3 × monthly IVI 2 mg/0.05 mL + SML within 1 week postloading dose
Furashova et al., 2020 [201]	Ranibizumab	810 nm	‐ 15% 200 ms	3 × monthly IVI 0.5 mg/0.05 mL + SML 8, 12 weeks postloading dose
Kanar et al., 2020 [202]	Aflibercept	577 nm	160 µm 5% 20 ms	3 × monthly IVI 2 mg/0.05 mL + SML at 1 month if CMT < 450 µm
Altınel et al., 2021 [203]	Bevacizumab	577 nm	160 µm 5% 200 ms	3 × monthly IVI 1.25 mg/0.05 mL + SML 4 weeks postloading dose if CMT < 400 µm
ElMatri et al., 2021 [195]	Bevacizumab	577 nm	200 µm 5% 200 ms	3 × monthly IVI 1.25 mg/ 0.05 mL + SML within 1 week postloading dose
Koushan et al., 2022 [196]	Aflibercept	532 nm	200 µm 10% 20 ms	1 × IVI 2.0 mg/0.05 mL + SML on the same day + SML 4 weekly
Bıçak et al., 2022 [204]	Ranibizumab	577 nm	165 µm 5% 200 ms	3 × monthly IVI 0.5 mg/0.05 mL + SML 4 weeks postloading dose, only once time

Abbreviations: BCVA, best corrected visual acuity; CMT, central macular thickness; IVI, intravitreal injection; SML, subthreshold micropulse laser; VEGF, vascular endothelial growth factor; ‐, unmentioned.

## 5 Diagnostic and Therapeutic Monitoring Innovations

DR is progressive and insidious, but it can be preventable and manageable. Early screening and intervention can reduce the risk of blindness by 56% [205]. Traditionally, the diagnosis and follow‐up of DR are based on retinal structural abnormalities and performed by fundus examination, color fundus photography, and so forth. However, these methods lack sufficient sensitivity for early detection, as they mainly rely on late‐stage hemorrhagic lesions, including large microaneurysms, hemorrhage, intraretinal microvascular abnormalities, or neovascularization [206]. Additionally, they provide only a single‐dimensional assessment and fail to reveal deeper pathological changes. This often produces a lag in diagnosis relative to actual pathophysiological changes, and therefore the earliest window for intervention is missed.

The development of imaging technology and artificial intelligence (AI) has shifted the diagnosis and therapeutic monitoring of DR from passive, general morphological grading to proactive, individualized risk prediction and precise management. The future DR monitoring model is expected to integrate multimodal precision diagnosis, AI‐driven broad screening, and biomarker‐based early warning systems.

### 5.1 Multidimensional Extension of Diagnostic Dimensions

Seven overlapping 30° fundus photographs that cover the posterior pole and mid‐periphery are known as standard seven‐field fundus photography, and for a long time, they have been considered the gold standard for the diagnosis and grading of DR [207]. However, the above process requires active cooperation from the patient and high‐level skills from the technician, and some peripheral retinal lesions may still go undetected. These issues have limited its widespread use in DR screening environments. With the development of digital and imaging technology, ultra‐wide‐field fundus fluorescein angiography (UWFA) and optical coherence tomography (OCT) have begun to be applied in the examination and monitoring of DR. UWFA extends the range of traditional fundus photography by covering up to 200° of the retina in a single frame, which is 3.2 times that of traditional seven‐field fundus photography, and includes visualization of the peripheral retina [208]. OCT is now being used around the world for diagnosis and monitoring of many retinal diseases, and noninvasive high‐resolution cross‐sectional images have begun to be obtained for DME as well [209]. Compared to traditional fundus photography, it can more sensitively visualize macular edema, with edema localization and volume quantification. In addition to being the gold standard for monitoring DME treatment responses, OCT is also used for guiding anti‐VEGF or steroid therapy [210]. Optical coherence tomography angiography (OCTA) is a new complement to OCT that can visualize retinal and choroidal vessels without contrast agents, so it has been used for a long time in early detection of ischemia and quantification of neovascularization [211]. OCTA creates three‐dimensional images with high detail of retinal and choroidal blood vessels and provides information on vascular flow at different times, so it has shown good prospects for the development of DR diagnosis and monitoring [212].

### 5.2 AI Diagnostic System

With the use of large datasets and individualization models for prediction, AI in medical images will be applied to many large‐scale health examinations and real‐time monitoring. Through efficiently screening color fundus photographs and performing pixel‐level precise localization and quantitative analysis of early, subtle lesions in OCTA images, the diagnostic capabilities of AI far exceed those achievable by the naked eye [213]. In addition, AI diagnostic systems have shown great potential in enhancing medical decision‐making, expanding patient accessibility, and improving cost‐effectiveness. IDx‐DR (Digital Diagnostics, Coralville, Iowa, United States) is the first AI‐based diagnostic system that has been listed as a Class IIa medical device by the FDA for autonomous diagnosis of DR [214]. Two standard 45° pictures of the center of the macula and the optic disk are taken for diagnosis by the system, and referable DR can be identified with a detection accuracy close to that of manual grading [215]. At present, AI diagnostic systems are still based on two‐dimensional photography, and the lack of stereoscopic observations may affect the ability of these systems to detect macular edema and PDR fibrovascular changes. In the future, the research may focus on integrating AI diagnostics with multidimensional data such as UWFA, OCT, OCTA imaging, and whole‐body clinical data to build an all‐encompassing assessment model.

### 5.3 Emerging Frontiers in Molecular Diagnostics

The identification of diagnostic and predictive biomarkers that reflect the pathophysiology of DR to achieve ultra‐early warning and therapeutic monitoring is a cutting‐edge focus in this field [216]. Numerous studies have shown that specific molecules in blood, aqueous humor, or vitreous, such as inflammatory factors, oxidative stress products, angiogenic factors, and noncoding RNAs, have potential as biomarkers for identifying high‐risk individuals before visible retinal lesions appear [217, 218]. This heralds a revolutionary shift in diagnostic paradigms: from diagnosing existing diseases to preventing future diseases. Further, different biomarkers correspond to different pathological pathways, which may elucidate disease mechanisms and subtypes, thus providing a biological basis for precision treatment in the future [219]. In clinical use, biomarkers, particularly blood biomarkers, are convenient to obtain and can be frequently retested. This makes it possible to dynamically monitor disease activity and assess treatment response and therefore adjust treatment plans using real‐time molecular‐level feedback. However, due to the unclear correlation between circulating biomarkers and specific diseases and the difficulty of obtaining invasive biomarkers, most biomarkers are still in research stages or early stages of translation and are not yet widely used in routine clinical diagnosis.

## 6 Special Populations and Comorbidities

Over 40% of DME patients respond poorly to anti‐VEGF therapy [220], necessitating target switching or combination strategies. Management must also be tailored for special populations—pregnancy, children/adolescents, and the elderly—each with distinct risk profiles and treatment considerations. Poor‐responders are defined as those who fail to gain at least five ETDRS letters in BCVA or achieve a 20% or greater reduction in CMT after three consecutive anti‐VEGF injections [221]. In clinical practice, switching treatment targets or using a combination therapy is generally recommended for poor responders [91]. However, the number of pharmacological targets is limited. In addition to changing the type of anti‐VEGF agent, steroids, particularly long‐acting implants, are a preferred treatment. In the future, new drugs with different mechanisms of action (see Table 1) are expected to provide breakthroughs in treating refractory DME. Further, the simultaneous or sequential targeting of different pathological mechanisms of DR or combining anti‐VEGF drugs with steroids or with SML provides an opportunity for poor responders to overcome treatment bottlenecks and regain disease control.

Pregnancy is an independent risk factor for the onset and rapid progression of DR [222]. According to the Standards of Care in Diabetes—2024, all women with diabetes should undergo a comprehensive eye examination before planning pregnancy or early in the pregnancy. Depending on the severity of retinopathy, regular follow‐up appointments should be scheduled throughout pregnancy and for 1 year postpartum [223]. Substantial clinical evidence has confirmed the safety of laser photocoagulation during pregnancy and its effectiveness in reducing the risk of vision loss. If laser therapy is not feasible or is ineffective and vision becomes seriously threatened, anti‐VEGF agents can be used as appropriate following a risk–benefit assessment. Steroid therapy is generally not recommended to avoid interfering with blood glucose and blood pressure control during pregnancy [224].

In children and adolescents, DR is mainly caused by Type 1 diabetes, and the likelihood of developing is correlated with the duration of the disease and puberty. Adolescents with a long history of the disease (> 5 years) and poor glycemic control are more prone to DR than adults and have a very high lifetime risk of vision‐threatening lesions. The patients should follow the screening instructions strictly and achieve optimal glycemic and blood pressure control from the time of diagnosis [225].

Elderly patients often have many chronic diseases and serious eye problems; they are not good at taking regular medications either. Unlike the goal of pursuing optimal vision in younger patients, for older adults with DR, it is necessary to weigh the benefits of effective treatment against the risks of serious side effects. Anti‐VEGF agents are the most effective method for improving vision. However, injection‐related risks must be considered, and long‐acting formulations should be prioritized to extend injection intervals [226]. Additionally, more weight should be given to the management of systemic complications in elderly patients, such as chronic kidney disease and cardiovascular disease, and personalized ophthalmological treatment plans need to be developed through multidisciplinary collaboration.

## 7 Future Therapeutic Frontiers

Many innovative DR therapies have been proposed [22, 23, 24, 227], yet their narrow perspective and insufficient clinical evidence hinder clinical adoption. To more comprehensively and accurately reflects the actual, achievable research directions and frontiers in DR therapy, we conducted a systematic, large‐scale bibliometric study using multiple methodologies. The macrolevel layout capabilities (clustering map) of VOSviewer combined with the microlevel tracking capabilities (timeline view, burst analysis) of CiteSpace [228, 229, 230], enabled a quick understanding of the overall structure and distribution of the major research forces in the field, and supported the precise capture of new research directions and potential breakthroughs.

We analyzed 14,875 publications from the Web of Science Core Collection (WoSCC) and examined multiple aspects of the dataset, including publication outputs, countries, institutions, authors, co‐cited journals, co‐cited references, keywords, and funding (see Supporting Information for more details). From 2014 to 2024, the volume of publications gradually increased, peaking in 2021 and maintaining a high level to the present time. Throughout the global research history of DR therapy, the United States, China (Mainland), and England have led in research output, academic impact, and international collaboration. Professor Tien Yin Wong of Tsinghua University emerged as the most influential scholar in terms of individual academic contributions, with substantial research output and high‐quality outcomes. His research has mainly focused on ophthalmic diseases, particularly retinal disorder mechanisms, innovative applications of AI and digital technologies, and interdisciplinary collaboration to advance medical translation. *IOVS* and *Ophthalmology* were identified as the core journals in DR therapy field.

**FIGURE 5:**
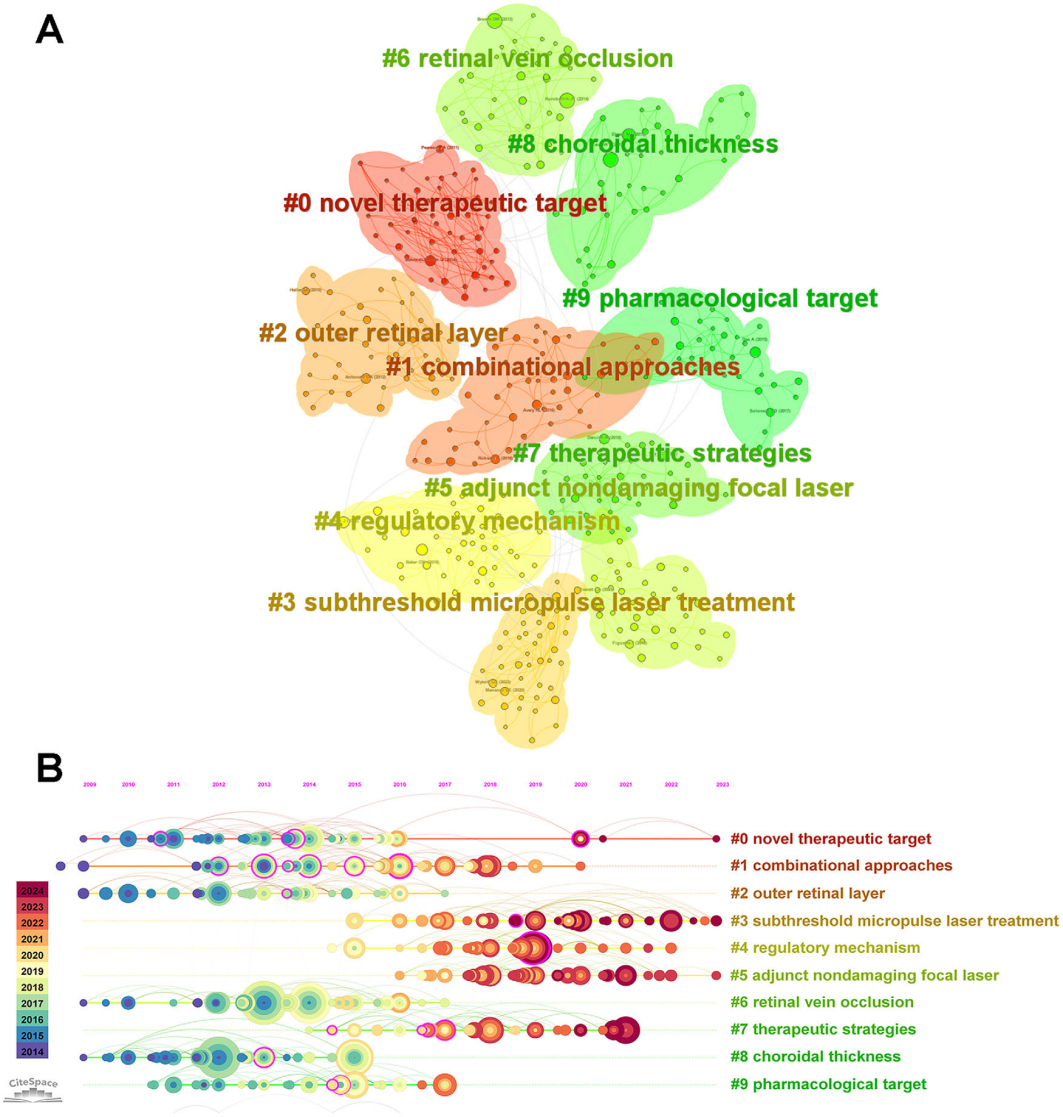
Co‐citation analysis of references in the field of diabetic retinopathy (DR) therapy from 2014 to 2024. (A) Cluster analysis of co‐cited references. (B) Timeline distribution of the top 10 clusters of co‐cited references. Novel therapeutic target and subthreshold micropulse laser treatment stand out as research hotspots and future trends in DR therapy. Created with CiteSpace (version 6.4.R1).

A co‐cited reference is a reference that is cited by many papers at the same time and can be considered an important research basis for that field [231]. Among the top 15 co‐cited references, 13 of them have used intravitreal anti‐VEGF agents for treatment; therefore, it can be seen that anti‐VEGF therapy is a typical way to manage DR. The most commonly cited co‐cited reference is the multicenter RCT by Wells and others in 2015, “Aflibercept, bevacizumab or ranibizumab for diabetic macular oedema” [232]. Most patients with DME showed a good improvement in visual acuity after receiving intravitreal injections of aflibercept, bevacizumab or ranibizumab, and aflibercept was more effective than the others in improving the vision of people with poor baseline vision. These landmark findings demonstrated the clinical efficacy of anti‐VEGF therapy, leading to a paradigm shift in DR research. In addition, eight of the 16 references with significant citation bursts also recommended anti‐VEGF therapy as the first‐line treatment for DR, and six investigated the clinical safety and effectiveness of macular laser photocoagulation and SML as adjuvant therapy for DR. This showed that over the past decade, attention to targeted biological agents (such as anti‐VEGF drugs) and SML therapy has been rising; at the same time, there have been developments in the pharmacological targets, clinical application of new drugs, and research on SML‐assisted treatment regimens. To gain insight into the distribution of these references both temporally and spatially, a co‐citation clustering view and a timeline map were created using CiteSpace (Figure 5). Novel therapeutic targets and SML therapy stood out as research hotspots and future trends in DR therapy.

A keyword co‐occurrence analysis can help quickly identify research hotspots in a particular field. The network map of keywords was field‐normalized into four major clusters: (1) pathological mechanisms of DR; (2) therapeutic interventions of DR; (3) prevalence and risk factors of diabetes and diabetes‐related complications; and (4) diagnostic tests and ocular imaging technology for DR. These clusters collectively underscored that in research on therapeutic interventions for DR, early diagnosis and management of primary diseases should not be overlooked. Further, a keyword timeline view and keyword burst analysis indicated that, in addition to the current focus on anti‐VEGF therapy, therapeutic exploration was shifting from late‐stage vitrectomy interventions toward innovative targeted agents and SML therapy. These results were consistent with those of the reference co‐citation analysis.

However, several limitations of the bibliometric study should be recognized. First, we analyzed only English‐language publications indexed in the WoSCC. Second, despite the powerful analytical tools available in bibliometrics, they also face many challenges in standardizing author and institutional names. Third, tools such as CiteSpace and VOSviewer rely on user‐defined threshold parameters, and the lack of unified standards limits the reproducibility of results. Finally, bibliometrics relies on citation counts to measure academic influence. Although bibliometrics offers objectivity and quantifiability, it also has limitations, including disciplinary differences, self‐citation risk, and poor indicator stability. In the future, a more comprehensive evaluation system should be constructed by incorporating alternative indicators, such as peer review, academic conferences, and interdisciplinary cooperation.

## 8 Challenges and Global Perspectives

The global perspectives of DR therapy are anticipated to transition toward multitarget strategies, sustained delivery platforms, and early prevention. On this basis, anti‐VEGF‐based targeted biologics have become a dominant research direction in DR therapy, with the aim of shifting management from late‐stage rescue to early, long‐acting intervention. Further, nanomedicine, TCM, and SML are emerging as complementary approaches for DR, offering substantial application potential, though they still face challenges in clinical translation.

Targeted biological agents, primarily anti‐VEGF agents, have emerged as the main research direction in DR therapy and hold great promise. They are expected to shift DR therapy from passive intervention for late‐stage complications to active management of the entire disease course, with the goal of blocking disease progression earlier, more persistently, and more effectively. However, the clinical translation of innovative targeted biological agents still faces numerous challenges, including poor efficacy and response due to the inherent limitations of single targets, challenges in drug delivery and the intraocular barrier, and the associated economic burden. In the future, basic and applied research should urgently be carried out in the following three areas: First, as for core targets, it should be expanded from a single VEGF target to a multitarget combination; Second, in terms of the route of administration, it needs to be changed from frequent intraocular injections to long‐acting sustained‐release; Third, regarding treatment strategy, it should move away from rescuing late‐stage cases to early intervention and prevention.

Considering that optimal therapeutic agents must overcome bioavailability challenges, innovation in drug delivery routes is also a major research direction in DR therapy [127, 128]. Nanocarriers have demonstrated tremendous potential for overcoming the limitations of conventional drugs, such as poor targeting, short half‐lives, and limited administration routes. However, their clinical application still faces multiple obstacles, such as high production costs, potential biotoxicity and immunogenicity, and limited targeting capabilities. The inspiring findings of our previous research indicated that ultrasound‐mediated microbubbles can safely and effectively treat retinal artery occlusion and confer neuroprotection [233], suggesting that noninvasive physical stimuli (e.g., ultrasound, magnetic fields [234], light irradiation [235]) can enhance the precision and efficacy of nanomedicine delivery. Hence, advancing nanomedicine in clinical practice requires strengthening interdisciplinary collaboration across materials science, biology, toxicology, and physics. With the continued refinement of fabrication techniques and accumulation of clinical evidence, nanomedicine is poised to become a cornerstone of DR therapy.

In contrast to the current targeted biological agents that primarily target DME and PDR, TCM can exert holistic regulatory effects on DR through its multiple components and multitarget pharmacological mechanisms; therefore, it is suitable for the early prevention and treatment of DR [115, 116]. However, most TCM studies have been performed using in vitro or animal models that do not fully recapitulate human metabolic processes or pharmacokinetics. Further rigorous mechanistic studies and well‐designed clinical trials are urgently needed to validate the efficacy, safety, and potential of integrating TCM into modern therapeutic frameworks.

SML is the first or second choice for DME and moderate to severe NPDR. Although very safe, it sacrifices some therapeutic effect, resulting in limited clinical use. There is no consensus among doctors around the optimal dosage or combined treatment of SML, so they must base their decisions on their own experience. Future efforts should focus on establishing operational consensus and individualized treatment plans based on clinical evidence and experience sharing.

## 9 Conclusions

**FIGURE 6:**
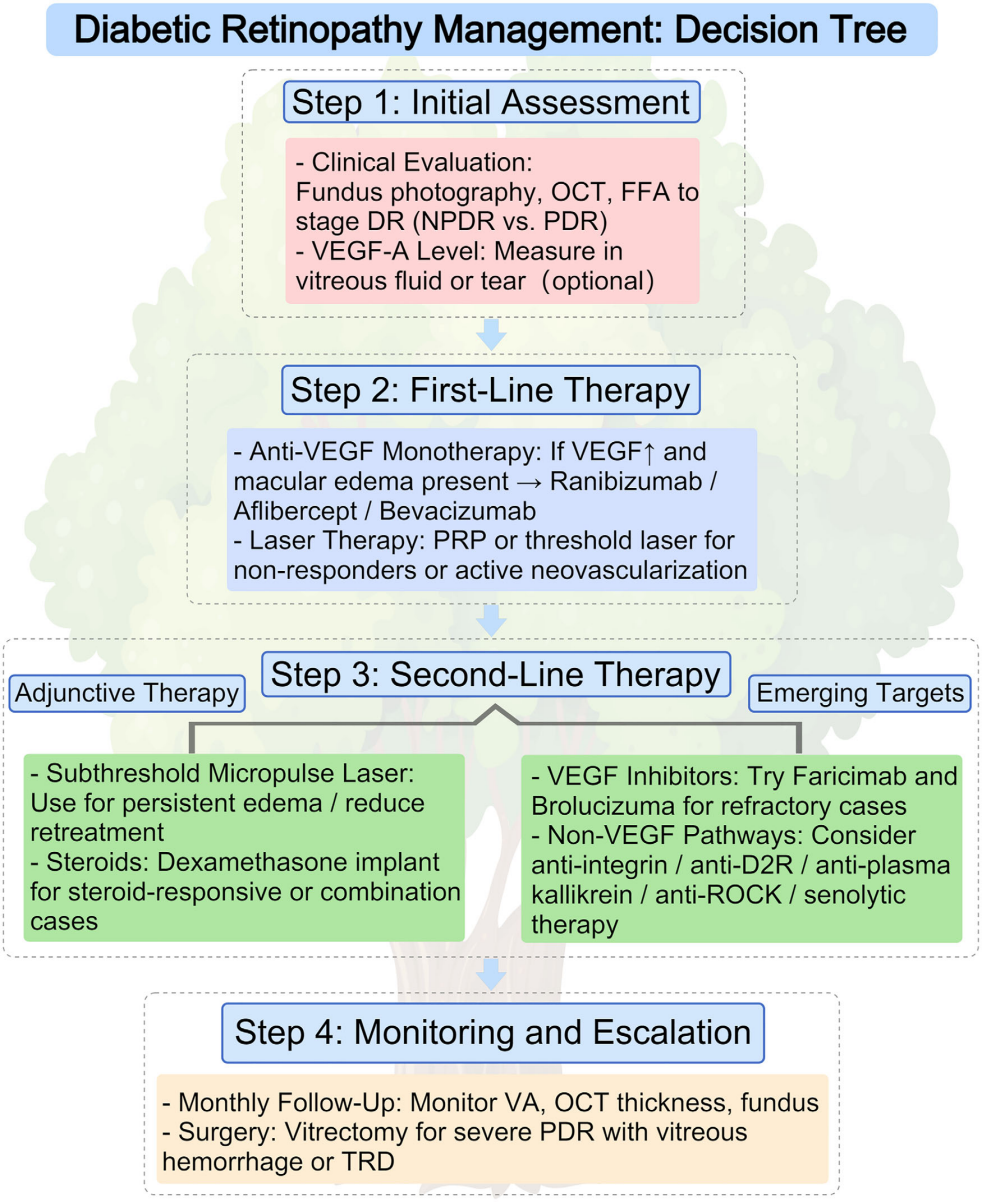
Clinical decision schematic for diabetic retinopathy (DR). This diagram outlines the four‐step decision‐making pathway for DR management. (1) Initial assessment: DR staging and risk stratification; (2) First‐line therapy: Antivascular endothelial growth factor (VEGF) therapy and panretinal photocoagulation (PRP); (3) Second‐line therapy: Subthreshold micropulse laser (SML), steroids, and emerging targeted biological agents; (4) Monitoring and escalation: Monthly follow‐up, with vitrectomy for severe proliferative diabetic retinopathy (PDR). D2R, dopamine D2 receptor; FFA, fundus fluorescein angiography; NPDR, nonproliferative diabetic retinopathy; OCT, optical coherence tomography; ROCK, Rho/Rho‐associated protein kinase; TRD, traction retinal detachment; VA, visual acuity. Created with Medpeer.cn.

In light of the growing global incidence of DR, it is imperative for ophthalmologists and researchers in related fields to stay abreast of therapeutic advances and research trends. This review revealed a paradigm shift in DR therapy, from solely managing late‐stage vascular complications to addressing the entire disease spectrum with personalized precision medicine. The development of pharmacological targets, the clinical translation of long‐acting formulations, and the optimization of SML‐assisted treatment regimens are future trends in DR management with strong prospects. Clinicians should consider developing optimal treatment plans based on disease characteristics, patient prognosis, drug tolerance, and financial capacity to maximize efficacy while minimizing the economic burden. Promising candidate drugs in the clinical pipeline also merit increased attention to address cases of refractory DR. Figure 6 outlines a clinical decision schematic for DR. For basic scientists, enhancing interdisciplinary exchange and collaboration is essential for accelerating the development of multitarget biologics and sophisticated nanocarrier systems to ultimately realize the clinical translation of innovative therapeutic strategies.

## Author Contributions

All of the authors have made substantial contributions to this work. The study was conceived by Xinying Hu, Zhe Cha, Haiwei Xu, and Xuedong Xu. Haiwei Xu acquired the funding. Data curation and analysis were performed by Xinying Hu, Zhe Cha, Ao Lu, Wuping Xu, Xiaoqing Zhang, and Haiwei Xu. All of the authors conducted the investigation. Project administration and supervision were the responsibility of Xinying Hu, Zhe Cha, Xiaoqing Zhang, Sheng Miao, Haiwei Xu, and Xuedong Xu. Software was provided by Xinying Hu, Ao Lu, and Xuedong Xu. Visualization was performed by Xinying Hu, Zhe Cha, Ao Lu, Wuping Xu, and Haiwei Xu. The original draft was written by Xinying Hu and Wuping Xu. All of the authors participated in reviewing and editing the manuscript and have approved the final version.

## Funding

The author(s) declare that financial support was received for the research and/or publication of this article. This work was supported by the National Key Research and Development Program of China (Grant No. 2024YFA1108700) and the Research Project of Jiangyin Municipal Health Commission (Grant No. Z202403).

## Ethics Statement

The authors have nothing to report.

## Conflicts of Interest

The authors declare no conflicts of interest.

## Supporting information




**TABLE S1**: Evidence summary on Chinese medicine monomers for DR.
**FIGURE S1**: Emission mode of conventional laser and SML.

## Data Availability

The data that support the findings of this study are available from the corresponding author upon reasonable request.
