# Supplementary material for: Current Landscape and Future Perspectives of Diabetic Retinopathy Therapy: Pharmacological Targets, Precision Laser Technology, and Clinical Evidence
Source: MedComm (2020). 2026 Jul 7;7(7):e70849. doi: 10.1002/mco2.70849 (PMC13338632; doi:10.1002/mco2.70849)
Supplement: Supplementary file 1 — TABLE S1: Evidence summary on Chinese medicine monomers for DR. FIGURE S1: Emission mode of conventional laser and SML. [file MCO2-7-e70849-s001.docx]

**Current Landscape and Future Perspectives of** **Diabetic Retinopathy Therapy: Pharmacological Targets, Precision Laser Technology, and Clinical Evidence**

Running head: Advances in diabetic retinopathy therapy

Xinying Hu^1,2#^, Zhe Cha^2#^, Ao Lu^3^, Wuping Xu^1^, Xiaoqing Zhang^1^, Sheng Miao^1^, Haiwei Xu^2*^, Xuedong Xu^1*^

^1^Department of Ophthalmology, Jiangyin People's Hospital Affiliated to Nantong University, Jiangyin, 214400, Jiangsu, PR China.

^2^Department of Ophthalmology, Southwest Hospital/Southwest Eye Hospital, Third Military Medical University (Amy Medical University), Chongqing Key Laboratory of Visual Injury and Regeneration, Chongqing, 400038, PR China.

^3^Department of Ophthalmology, The First Affiliated Hospital of Chongqing Medical University, Chongqing, 400038, PR China.

^#^ Xinying Hu and Zhe Cha contributed equally to this work.

* Correspondence:

Haiwei Xu, Tel: +862365426424, Email address: xuhaiwei@tmmu.edu.cn.

Xuedong Xu, Tel: +8613815135005, Email address: dr_xuxuedong@163.com

**Bibliometric analysis**

Export data from WOSCC is publicly available on Zenodo (<https://zenodo.org/records/17751933>)

# Methods

**Data sources and search strategy**

The overall workflow is shown in Fig. S1. Data were retrieved from the Web of Science Core Collection (WoSCC; Science Citation Index Expanded) on October 16, 2024. We limited the search to English language, peer-reviewed publications from January 1, 2014, to October 16, 2024. Two authors performed independent searches to enhance the accuracy and reproducibility of the search results. To generate the most precise dataset for subsequent analysis, we used the following topic search (TS): (TS=(diabetic retinopathy) OR TS=(diabetic macular edema)) AND (TS=(therap*) OR TS=(treat*) OR TS=(cure) OR TS=(naturopathy) OR TS=(physiotherapy) OR TS=(medic*) OR TS=(drug*) OR TS=(pharma*) OR TS=(laser) OR TS=(photocoagulation) OR TS=(surg*) OR TS=(vitrectomy) OR TS=(inject*)). After excluding 1,426 non-peer-reviewed items (e.g., meeting abstracts, letters, book chapters, and editorial materials) and removing duplicates, the final dataset comprised 12,158 original research articles and 2,717 review articles.


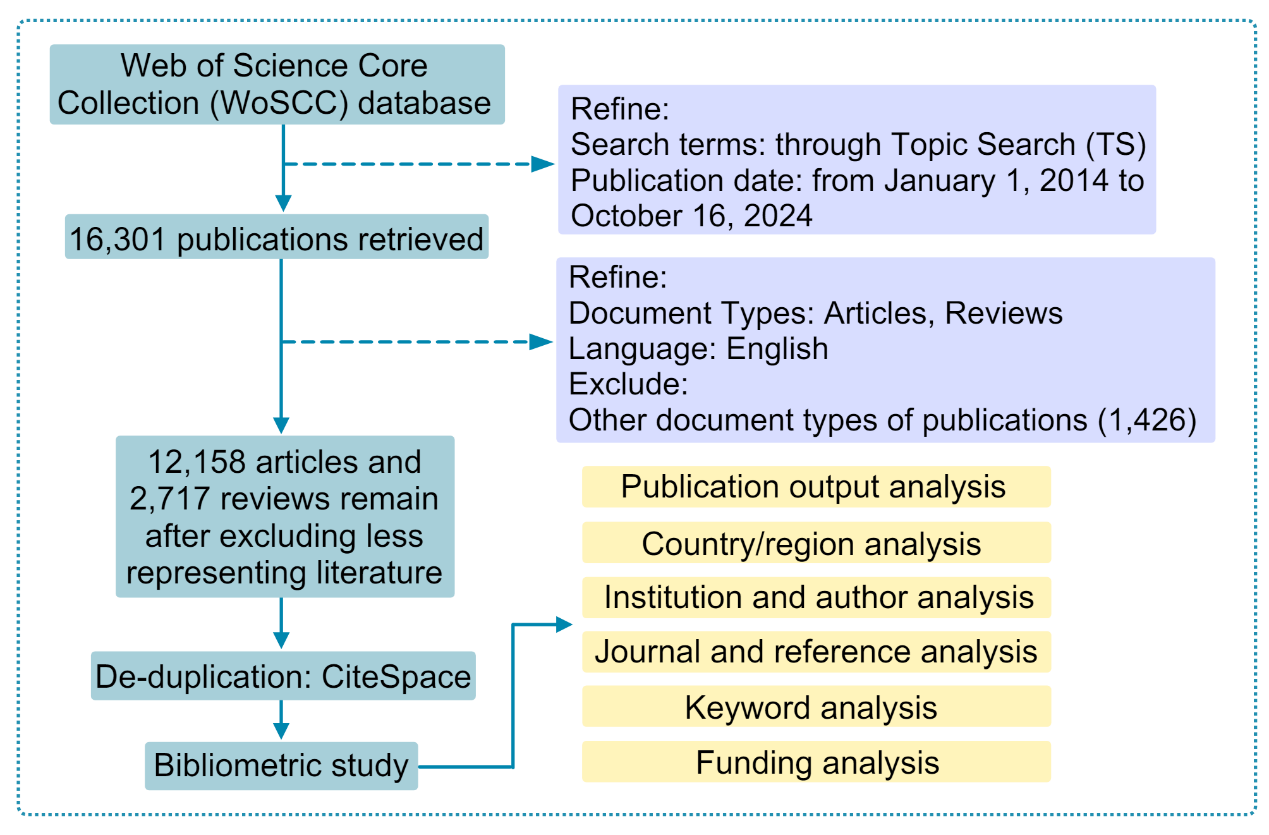


**Fig. S1.** The overall schematic diagram of this study.

**Data analysis and visualization**

Data on the annual number of publications and citations, countries (regions), institutions, authors, journals, and funding sources were extracted from the WoSCC. The h-indices, defined as the number of publications by an author cited at least h times, were also collected to evaluate both the quantity and quality of academic output [57]. Quantitative analyses and basic visualizations were performed using Microsoft Excel (Microsoft Corporation, Redmond, Washington, United States, version 16.57). We used VOSviewer (Leiden University, Leiden, Netherlands, version 1.6.20), a widely used tool for visualizing co-authorship, co-citation, and keyword co-occurrence relationships, to construct bibliometric networks [58, 59]. VOSviewer-generated maps represent entities, such as countries (regions), authors, journals, and keywords, as nodes. The node size reflects the number of publications or occurrences, the node color denotes clustering or classification, and the thickness of the line connecting nodes indicates the strength of collaboration or co-citation relationships between the node items [60]. In addition, CiteSpace (Drexel University, Philadelphia, Pennsylvania, United States, version 6.4.R1) was used to identify emerging trends and research frontiers by performing a burst analysis and timeline mapping of co-cited references and keywords [61]. Thresholds were dynamically established based on research objectives, data characteristics, and domain experience. The VOSviewer and CiteSpace results were integrated to generate a robust, data-driven overview of DR therapy research trends during the study period.

**Literature review**

In a bibliometric analysis, frequently cited references and keywords are crucial for identifying the research focus and future directions of a topic [1]. Based on analyses of reference co-citations and keyword co-occurrences supplemented by additional information from other sources (academic conferences, funding support, interdisciplinary cooperation), we identified that the emerging trends in DR therapy were targeted biological agents and SML therapy. Then, a detailed description and in-depth discussion of the two research hotspots from a microscopic perspective were established. Specifically, the subsequent literature review focused on cutting-edge advances and representative outcomes in these two research areas, with an emphasis on clinical candidate drugs and their clinical trial results. Furthermore, the mechanisms, targets, and efficacy of mainstream treatment methods were summarized and compared to provide comprehensive insights into the current status and prospects of DR therapies.

# Results

# Publications and annual growth

A total of 14,875 publications related to DR therapy were identified, comprising 12,158 original research articles (81.73%) and 2,717 reviews (18.27%). The annual volume of publications steadily increased during the study period, as shown in Fig. S2A, increasing from an average of 1,099.7 publications per year in 2014–2019 to 1,655.4 publications per year in 2020–2024 (through October 16, 2024). The number of publications peaked in 2021 at 1,845 and has remained high since then. The sustained increase in the publication rate indicates continued global attention on DR therapy research.

# Country and region analysis

Over the past decade, 138 countries and regions have contributed to the DR therapy research literature. The top 15 countries and regions in terms of publication volume are shown in Fig. S2B. Mainland China dominated the list with 3,809 publications (25.61%), and the United States (USA) ranked second (3,748 publications, 25.20%). To further assess the most influential countries and regions, the h-index and link strength were also evaluated as critical indicators. As shown in Fig. S2C, an international collaboration network of the countries and regions was constructed by including those with ≥ 200 publications (24/138). The USA held the leading position in DR therapy based on its high academic impact (h-index = 145) and extensive collaboration network (total link strength = 2149). This explained why mainland China had less influence than the USA, despite having the largest number of publications. Notably, although Singapore had a relatively modest publication output, it showed significant academic influence, with an impressive citation rate of 61.30 citations per publication.


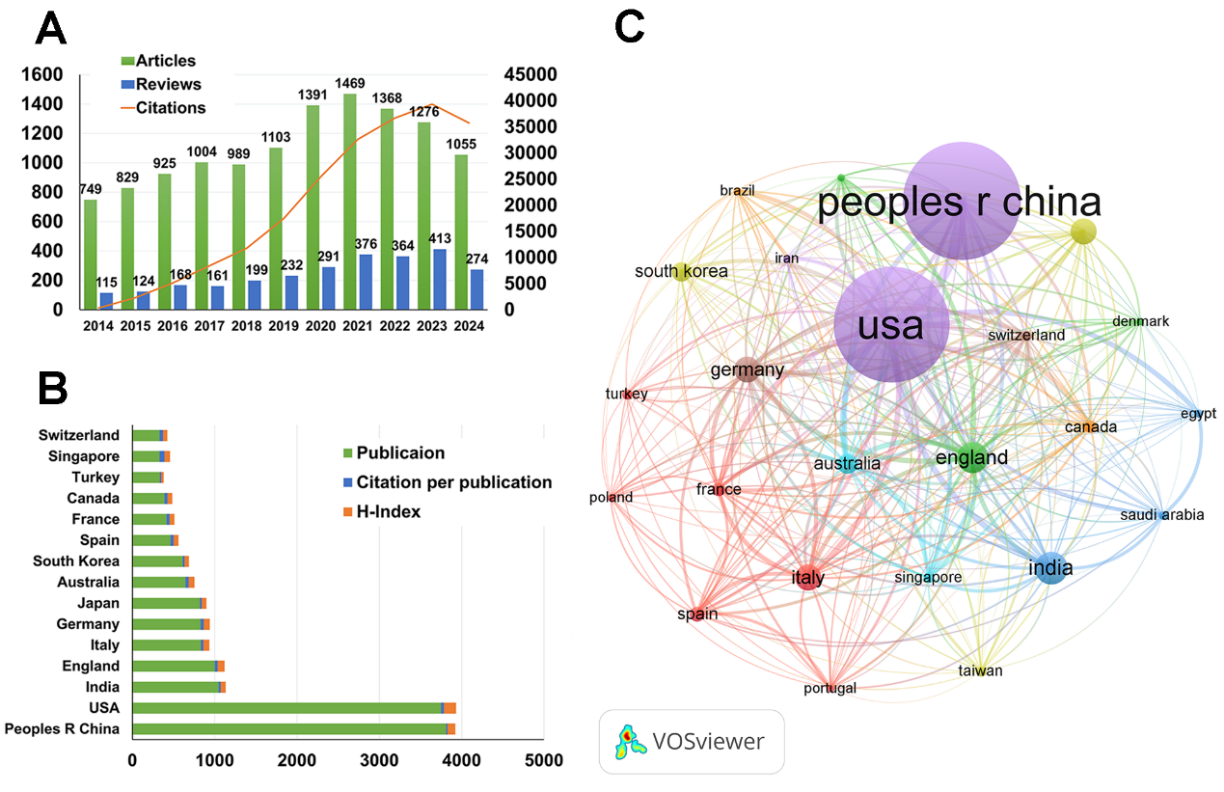


**Fig. S2.** Publication characteristics and regional distribution analysis.

(A) The illustration of the publication characteristic from Jan 1, 2014 to Oct 16, 2024. (B) The most contributing countries/regions in DR therapy field. (C) International collaboration among countries/regions in DR therapy field.

Peoples R China means the mainland China.

# Institution and author analysis

During the timeframe of the study, 55,503 authors from 12,017 institutions were involved in DR therapy. The top 15 most productive institutions were located in England, the USA, mainland China, Singapore, Egypt, and Australia. The University of London led with the most publications (484), followed by University College London (398), University of California System (380), and Harvard University (376), as shown in Fig. S3A. Although Johns Hopkins University ranked only eighth in publication count, it had the highest h-index (66) among the top institutions, indicating a strong academic influence and research impact on DR therapy. Similarly, Harvard University, University of London, and Johns Hopkins Medicine also boasted high h-indexes, demonstrating their substantial contributions to DR therapy research.

The top 15 most productive authors are shown in Fig. S3B (see Tab. S1 for details). The most prolific authors were Sobha Sivaprasad (Moorfields Eye Hospital, NHS Foundation Trust) and Tien Yin Wong (Tsinghua University), each with 107 publications, followed by Francesco Bandello (97) and Tunde Peto (74). Sobha Sivaprasad, despite ranking first in publication count, had citations per publication and h-index values that were relatively low. In contrast, Tien Yin Wong achieved the highest citations per publication (125.52) and h-index (49), signifying considerable contributions to DR therapy research in both quality and quantity.

**Tab. S1.** The top 15 productive authors in the DR therapy field.

| **Rank** | **Author** | **Institutes** | **Publication** | **Citation** | **Citation *per* publication** | **H-Index** |
| --- | --- | --- | --- | --- | --- | --- |
| 1 | Sivaprasad, Sobha | Moorfields Eye Hospital NHS Foundation Trust | 107 | 2047 | 19.13 | 25 |
| 2 | Wong, Tien Yin | Tsinghua University | 107 | 13431 | 125.52 | 49 |
| 3 | Bandello, Francesco | University of Calgary | 97 | 3083 | 31.78 | 24 |
| 4 | Peto, Tunde | The Queen's University Belfast | 74 | 4191 | 56.64 | 26 |
| 5 | Chhablani, Jay | University of Pittsburgh | 73 | 1332 | 18.25 | 19 |
| 6 | Wykoff, Charles C | Royal Victorian Eye & Ear Hospital | 69 | 3157 | 45.75 | 30 |
| 7 | Sun,  Jennifer K | Harvard Medical School | 66 | 7205 | 109.17 | 36 |
| 8 | Raman, Rajiv | Anglia Ruskin University | 64 | 5235 | 81.8 | 16 |
| 9 | Simo, Rafael | Autonomous University of Barcelona | 64 | 5238 | 81.84 | 28 |
| 10 | Schmidt-Erfurth, Ursula | Medical University of Vienna | 61 | 4019 | 65.89 | 25 |
| 11 | Sadda, SriniVas R | University of California Los Angeles | 54 | 1820 | 33.7 | 21 |
| 12 | Singh,  Rishi P | Cleveland Clinic Foundation | 54 | 890 | 16.48 | 14 |
| 13 | Loewenstein, Anat | Tel Aviv University | 53 | 2824 | 53.28 | 26 |
| 14 | Keane, Pearse | University College London | 51 | 3401 | 66.69 | 27 |
| 15 | Grauslund, Jakob | University of Southern Denmark | 51 | 864 | 16.94 | 16 |

Ranked by the number of publications

A co-authorship map of authors with ≥ 30 publications (55/55,503) was constructed, as shown in Fig. S3C. The map revealed five distinct clusters, primarily centered on the leading authors listed in Fig. S3B. These clusters indicated strong internal academic cooperation, with the most tightly connected cluster led by Sobha Sivaprasad (Moorfields Eye Hospital, NHS Foundation Trust). However, inter-cluster collaboration was relatively limited.

# Co-citation analysis of journals

In our study, 1678 journals were identified that published DR therapy-related research. As detailed in Tab. S2, *Investigative Ophthalmology & Visual Science (IOVS)* had the highest number of publications (425), followed by *Retina—The Journal of Retinal and Vitreous Medicines* (406) and *PLOS One* (361), all of which are USA-based journals. Although *Ophthalmology* ranked only 13th in terms of publication volume, it had the highest number of total citations at 17,887 and an h-index of 70, followed by *IOVS* (11,462 citations; h-index = 54), and *Retina—The Journal of Retinal and Vitreous Medicines* (10,113 citations; h-index = 51). *Ophthalmology* had the highest impact factor among all the journals included in the study.

**Tab. S2.** The top 15 productive journals in DR therapy field.

| **Rank** | **Journal**  **(Publisher country)** | **Publication** | **Citation** | **Citation *per* publication** | **H-Index** | **IF2023** |
| --- | --- | --- | --- | --- | --- | --- |
|  | Investigative Ophthalmology Visual Science (USA) | 425 | 11462 | 26.97 | 54 | 4.4 |
| 2 | Retina: the Journal of Retinal and Vitreous Diseases (USA) | 406 | 10113 | 24.91 | 51 | 3.3 |
| 3 | Plos One (USA) | 361 | 8120 | 22.49 | 46 | 3.7 |
| 4 | Scientific Reports (England) | 289 | 4905 | 16.97 | 35 | 4.6 |
| 5 | Graefes Archive for Clinical and Experimental Ophthalmology (USA) | 263 | 3860 | 14.68 | 30 | 2.7 |
| 6 | International Journal of Ophthalmology (China) | 236 | 2148 | 9.10 | 22 | 1.4 |
| 7 | Eye (England) | 228 | 4720 | 20.70 | 36 | 3.9 |
| 8 | BMC Ophthalmology (England) | 221 | 2278 | 10.31 | 25 | 2.0 |
| 9 | International Journal of Molecular Sciences (Switzerland) | 221 | 4981 | 22.54 | 33 | 6.2 |
| 10 | European Journal of Ophthalmology (Italy) | 214 | 1673 | 7.82 | 20 | 1.7 |
| 11 | Acta Ophthalmologica (Denmark) | 211 | 3617 | 17.14 | 29 | 3.4 |
| 12 | India Journal of Ophthalmology (India) | 204 | 1783 | 8.74 | 20 | 3.1 |
| 13 | Ophthalmology (USA) | 199 | 17887 | 89.88 | 70 | 13.7 |
| 14 | Experimental Eye Research (USA) | 190 | 3673 | 19.33 | 33 | 3.4 |
| 15 | JAMA Ophthalmology (USA) | 187 | 9736 | 52.06 | 58 | 8.1 |

Ranked by the number of publications

Co-citation occurs when two journals are both cited by a different publication [2]. Co-citation analysis, a method derived from bibliometrics, is a means of field normalization and hub location identification of cross-disciplinary networks [2, 3]. In this study, we identified 37,702 journal co-citations, which yielded a network of 905 co-cited journals after removing journals with less than 100 citations. *IOVS* was the most frequently co-cited journal, with a total co-citation count of 38,510, followed by *Ophthalmology* with 38,344 co-citations and *American Journal of Ophthalmology* with 16,135 co-citations. Fig. S3D shows the co-cited journals grouped into four major clusters, each distinguished by color and a thematic focus. The red cluster primarily represents journals publishing basic research studies, such as the pathogenesis and pharmacological mechanisms of DR. The green cluster highlights interdisciplinary links, with *Diabetes Care* prominent in this cluster*,* indicating that DR therapy is a concern across ophthalmology, diabetology, and metabolic disease research. The blue cluster includes journals with a strong clinical and translational focus, including *IOVS,* *Ophthalmology,* and *American Journal of Ophthalmology*. The yellow cluster mainly comprises journals related to medical imaging sciences and photographic techniques.


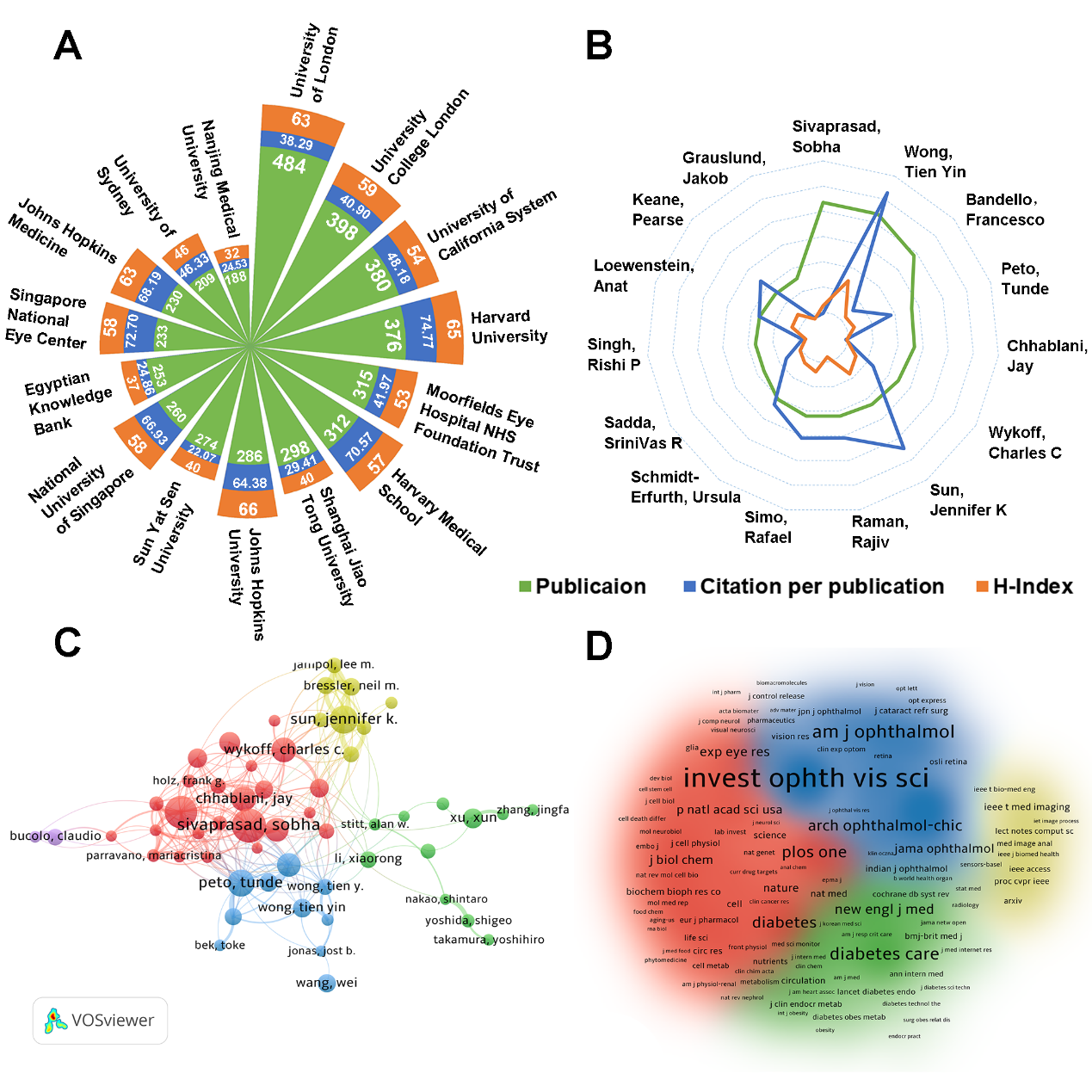


**Fig. S3.** Institutions/authors/co-cited journals analysis.

(A) The most contributing institutions in DR therapy field. (B) The most contributing authors in DR therapy field. (C) Cluster analysis of collaboration among authors in DR therapy field. (D) Co-citation density network of the journals in DR therapy field.

# Co-cited references and references with citation bursts

To gain a deeper understanding of the core literature and interdisciplinary connections in DR therapy research, we conducted a co-citation analysis of the references of each article. A co-cited reference refers to a publication that is cited by multiple articles, indicating its significance and influence within a research area [4]. Among the top 15 co-cited references listed in Tab. S3, 10 references were published in *Ophthalmology*. The rest were from *New England Journal of Medicine*, *Journal of The American Medical Association* (*JAMA)*, *Diabetes Care*, *Lancet*, and *JAMA Ophthalmology*. A report on a multicenter randomized controlled trial, “Aflibercept, bevacizumab, or ranibizumab for diabetic macular edema,” in the *New England Journal of Medicine* (IF 2023 = 158.5) was the most frequently co-cited study. This landmark study showed that all three anti-VEGF agents significantly improved visual acuity in patients with DME, with aflibercept showing superior efficacy in those with poorer baseline vision [5]. The second most co-cited reference was “Ranibizumab for diabetic macular edema: Results from two phase III randomized trials: RISE and RIDE,” published in *Ophthalmology* (IF 2023 = 13.7). This pivotal phase III clinical trial validated the efficacy and safety of intravitreal ranibizumab (anti-VEGF treatment) in patients with DME, marking a major milestone in the clinical treatment of DME and contributing significantly to the development of anti-VEGF therapy over the past decade [6]. Moreover, 13 of the top 15 co-cited references were focused on intravitreal anti-VEGF agents, highlighting the dominant role of anti-VEGF therapy in DR treatment.

**Tab. S3.** The top 15 co-cited references in the DR therapy field.

| **Rank** | **Author** | **Year** | **Journal** | **Title** |
| --- | --- | --- | --- | --- |
| 1 | Wells,  John A | 2015 | *New England Journal of Medicine* | Aflibercept, bevacizumab, or ranibizumab for diabetic macular edema |
| 2 | Nguyen, Quan Dong | 2012 | *Ophthalmology* | Ranibizumab for diabetic macular edema: results from 2 phase III randomized trials: RISE and RIDE |
| 3 | Gross, Jeffrey G | 2015 | *Jama-Journal of The American Medical Association* | Panretinal Photocoagulation vs Intravitreous Ranibizumab for Proliferative Diabetic Retinopathy: A Randomized Clinical Trial |
| 4 | Boyer, David S | 2014 | *Ophthalmology* | Three-year, randomized, sham-controlled trial of dexamethasone intravitreal implant in patients with diabetic macular edema |
| 5 | Mitchell, Paul | 2011 | *Ophthalmology* | The RESTORE study: ranibizumab monotherapy or combined with laser versus laser monotherapy for diabetic macular edema |
| 6 | Brown, David M | 2013 | *Ophthalmology* | Long-term outcomes of ranibizumab therapy for diabetic macular edema: the 36-month results from two phase III trials: RISE and RIDE |
| 7 | Wells,  John A | 2016 | *Ophthalmology* | Aflibercept, Bevacizumab, or Ranibizumab for Diabetic Macular Edema: Two-Year Results from a Comparative Effectiveness Randomized Clinical Trial |
| 8 | Korobelnik, Jean-Francois | 2014 | *Ophthalmology* | Intravitreal aflibercept for diabetic macular edema |
| 9 | Yau,  Joanne W Y | 2012 | *Diabetes Care* | Global prevalence and major risk factors of diabetic retinopathy |
| 10 | Brown, David M | 2015 | *Ophthalmology* | Intravitreal Aflibercept for Diabetic Macular Edema: 100-Week Results from the VISTA and VIVID Studies |
| 11 | Elman, Michael J | 2012 | *Ophthalmology* | Intravitreal ranibizumab for diabetic macular edema with prompt versus deferred laser treatment: three-year randomized trial results |
| 12 | Elman, Michael J | 2015 | *Ophthalmology* | Intravitreal Ranibizumab for diabetic macular edema with prompt versus deferred laser treatment: 5-year randomized trial results |
| 13 | Sivaprasad, Sobha | 2017 | *Lancet* | Clinical efficacy of intravitreal aflibercept versus panretinal photocoagulation for best corrected visual acuity in patients with proliferative diabetic retinopathy at 52 weeks (CLARITY): a multicentre, single-blinded, randomised, controlled, phase 2b, non-inferiority trial |
| 14 | Elman, Michael J | 2011 | *Ophthalmology* | Expanded 2-year follow-up of ranibizumab plus prompt or deferred laser or triamcinolone plus prompt laser for diabetic macular edema |
| 15 | Rajendram, Ranjan | 2012 | *JAMA Ophthalmology* | A 2-year prospective randomized controlled trial of intravitreal bevacizumab or laser therapy (BOLT) in the management of diabetic macular edema: 24-month data: report 3 |

Ranked by frequency of co-cited references

To explore thematic structures within the reference network, a co-citation clustering map was generated. The top 10 clusters revolved around five primary thematic areas, with “novel therapeutic target” and “pharmacological target” reflecting the exploration of novel pharmacological targets and targeted biological agents in DR therapy. “Adjunct nondamaging focal laser” and “subthreshold micropulse laser treatment” represented advances in non-invasive physical therapies, particularly the evolution of micropulse laser technologies. “Combinational approaches” and “therapeutic strategies” captured the current clinical perspectives on integrated DR treatment regimens. The remaining four clusters included studies on the morphological and mechanistic aspects of DR.

A timeline visualization of the co-citation clusters illustrates the evolution of key DR therapy research themes over time. The “novel therapeutic target” cluster has persisted since 2009, reflecting the continued research in DR pharmacological target development and the clinical translation of targeted biological agents over nearly 15 years. The clusters “adjunct nondamaging focal laser” and “subthreshold micropulse laser treatment” first appeared in 2015 and have continued to gain traction, suggesting a growing interest in SML therapy as an alternative or complement to pharmacological interventions.

A citation burst analysis was performed in CiteSpace to identify references that experienced a surge in citations over a specific period. There were 16 articles that were identified with significant citation bursts and their key contributions are summarized in Tab. S4. Eight of the articles affirmed anti-VEGF therapy as a first-line treatment for DR, and six of the articles discussed the clinical safety and efficacy of macular laser photocoagulation and SML therapy as adjunctive therapy for DR. The citation burst analysis further substantiated that targeted biological agents and the clinical application of SML therapy have garnered increasing attention over the past decade.

**Tab. S4.** The major research contents of the 16 references with strong citation bursts.

| **Rank** | **Author** | **Burst** **duration** | **Burst strength** | **Major research contents** |
| --- | --- | --- | --- | --- |
| 1 | Nguyen QD | 2014-2017 | 24.78 | Evaluating the efficacy and safety of intravitreal ranibizumab in DME patients |
| 2 | Gross JG | 2017-2020 | 22.56 | Evaluating the noninferiority of intravitreous ranibizumab compared with PRP for visual acuity outcomes in patients with PDR |
| 3 | Wells JA | 2016-2020 | 20.98 | Comparing the relative efficacy and safety of intravitreal aflibercept, bevacizumab, and ranibizumab in the treatment of DME |
| 4 | Wells JA | 2017-2020 | 15.61 | 2-year results comparing anti-VEGF agents for center-involved DME using a standardized follow-up and retreatment regimen |
| 5 | Brown DM | 2015-2018 | 15.11 | Reporting 36-month outcomes of clinical trials of ranibizumab in DME |
| 6 | Korobelnik JF | 2016-2019 | 14.82 | A head-to-head comparison was performed between vascular endothelial growth factor blockade and laser for the treatment of DME |
| 7 | Brown DM | 2017-2020 | 14.79 | Comparing efficacy and safety of 2 dosing regimens of IAI with macular laser photocoagulation for DME |
| 8 | Boyer DS | 2016-2019 | 14.35 | Evaluating the safety and efficacy of dexamethasone intravitreal implant 0.7 and 0.35 mg in the treatment of patients with DME |
| 9 | Elman MJ | 2014-2017 | 13.55 | Reporting the 3-year follow-up results within a previously reported randomized trial evaluating prompt versus deferred (for ≥24 weeks) focal/grid laser treatment in eyes treated with intravitreal 0.5 mg ranibizumab for DME |
| 10 | Schmidt-Erfurth U | 2019-2022 | 13.31 | A review concluded that based on recent rigorous clinical trial results, laser photocoagulation is no longer recommended for the treatment of DME, and anti-VEGF therapy has become the first-line treatment option |
| 11 | Sivaprasad S | 2018-2022 | 13.18 | Comparing the 1-year safety and efficacy of PRP and intravitreal aflibercept for the treatment of PDR |
| 12 | Gross JG | 2019-2024 | 12.32 | Evaluating efficacy and safety of 0.5-mg intravitreous ranibizumab vs PRP over 5 years for PDR |
| 13 | Heier JS | 2018-2021 | 10.69 | Comparing efficacy and safety of IAI with macular laser photocoagulation for DME over 3 years |
| 14 | Elman MJ | 2016-2020 | 10.62 | Reporting 5-year results from a previously reported trial evaluating intravitreal 0.5 mg ranibizumab with prompt versus deferred (for ≥24 weeks) focal/grid laser treatment for DME |
| 15 | Do DV | 2014-2017 | 9.87 | Comparing different doses and dosing regimens of VEGF Trap-Eye with laser photocoagulation in eyes with DME |
| 16 | Daruich A | 2020-2024 | 8.9 | A comprehensive review on the current understanding of macular edema and its mechanisms opens perspectives to identify new preventive and therapeutic strategies for this sight-threatening condition |

Ranked by burst strength. Abbreviations: DME: diabetic macular edema, PRP: panretinal photocoagulation, PDR: proliferative diabetic retinopathy, VEGF: vascular endothelial growth factor, IAI: intravitreal aflibercept injection.

# Keyword analysis

A keyword co-occurrence analysis can rapidly identify research hotspots in a particular field. In this study, a total of 29,865 keywords were identified within the dataset. By focusing on the 612 keywords with a frequency of 35 or higher, a co-occurrence network was constructed, as shown in Fig. S4A. The keyword network was field-normalized into four major thematic clusters. The red cluster had the greatest number of keywords (238), which were primarily related to mechanisms of DR, including “endothelial growth factor,” “oxidative stress,” “angiogenesis,” and “apoptosis”. These topics have received sustained attention over the past decade. The green cluster included terms related to the clinical treatment of DR, particularly anti-VEGF therapy for DME. Keywords such as “diabetic macular edema,” “ranibizumab,” “bevacizumab,” and “injection” highlighted the extensive research and development of biologic therapeutics targeting DME in recent years. The blue cluster included terms associated with diabetes prevalence, risk factors, and disease management. Frequently occurring keywords included “prevalence,” “risk factors.” “complications,” “progression,” and “management,” suggesting that preventive management, patient education, and epidemiological research are active research topics. The yellow cluster focused on diagnostic imaging technologies and ocular imaging technology-related topics, including the keywords “optical coherence tomography,” “fluorescein angiography,” “classification,” “diagnosis,” and “artificial intelligence.” The presence of this cluster underscored the strong link between early diagnosis and treatment of DR, suggesting that a research focus is being maintained on the early diagnosis of DR in addition to therapeutic interventions.

To uncover additional temporal patterns, we used CiteSpace to generate a keyword timeline and conduct a keyword burst analysis. As shown in Fig. S4B, early research themes included “pars plana vitrectomy,” “vitreous hemorrhage,” “cataract,” “retinal vein occlusion,” and “risk factors.” Since 2019, newer topics have become prominent, including “optical coherence tomography angiography,” “bevacizumab,” and “subthreshold micropulse laser.” This indicates that, in addition to anti-VEGF therapy as a first-line treatment, the direction of DR therapy is shifting towards early intervention and SML therapy to prevent or delay late-stage DR-related complications.

A keyword burst analysis was conducted, and the 25 keywords with the strongest citation bursts are presented in Fig. S4C. In early phases of DR therapy research, “follow-up,” “deferred laser,” “triamcinolone acetonide,” “pattern scan laser,” and “intravitreal bevacizumab avastin” were emphasized. Over time, attention has shifted toward “anti-vascular endothelial growth factor,” “coherence tomography angiography,” “cataract surgery,” “subthreshold micropulse laser,” “risk factors,” “ranibizumab treatment,” and “intraocular pharmacokinetics,” which represent recent breakthroughs in DR therapy, with a clear trend toward precise imaging diagnostics, innovative targeted biological agents, and SML combination therapeutic modalities in DR management.


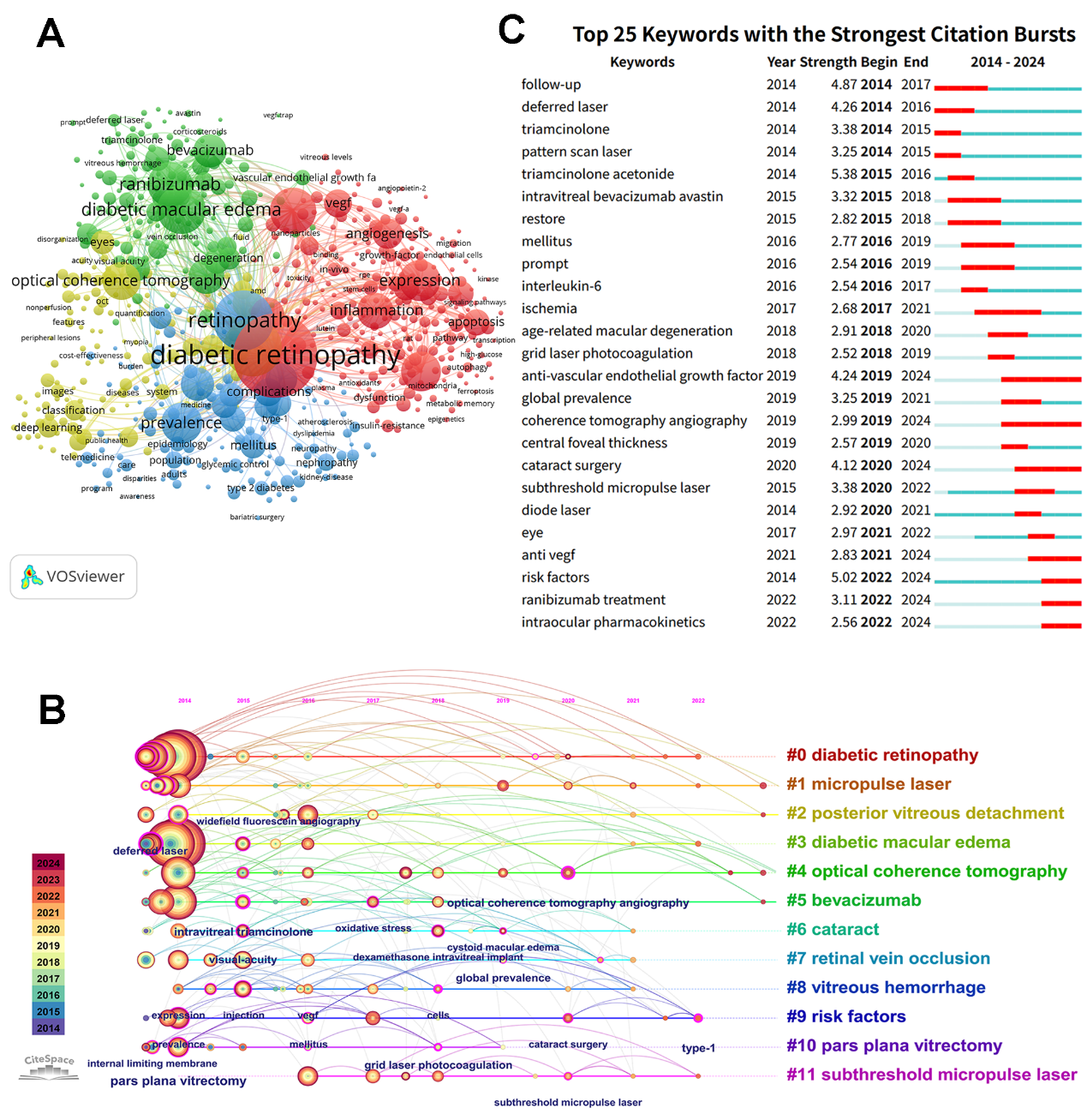


**Fig. S4.** Keyword analysis.

(A) The network map of keywords in DR therapy field. (B) Timeline distribution of keywords in DR therapy field. (C) The top 25 keywords with intense citation bursts in DR therapy field. A red bar indicates high citations in that year.

# Funding source analysis

The top 15 funding sources supporting research studies on DR therapy are shown in Fig. S5. Among them, four are in the USA, followed by three in Japan, and one each in mainland China, England, and Europe. The United States Department of Health and Human Services and the National Institutes of Health, both in the USA, made the strongest contributions based on publication count and research quality; both had an h-index of 106. Notably, five leading healthcare companies were also major contributors to DR therapy research output: Roche Holding, Novartis, AbbVie, Allergan, and Bayer AG. This suggests that targeted biological agents for DR therapy have significant clinical translation and commercial value potential.


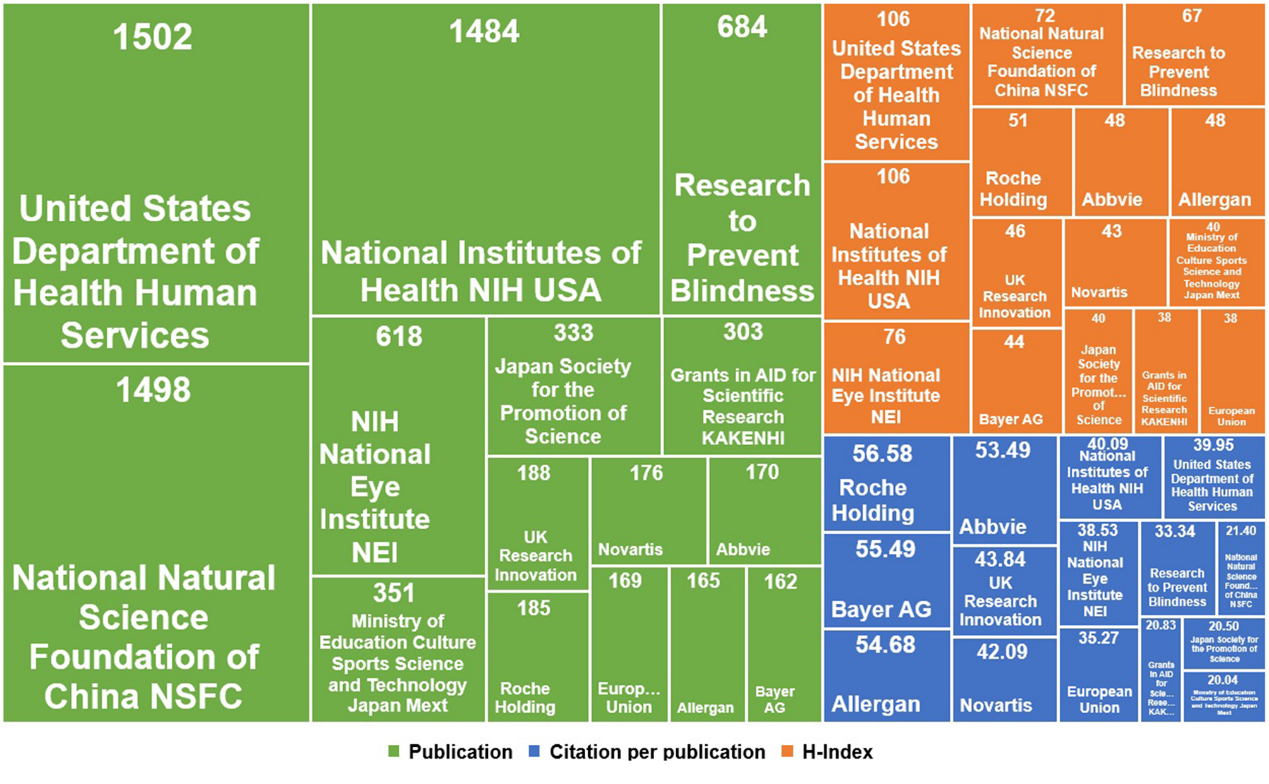


**Fig. S5.** The most contributing funding in DR therapy field.

**Tables**

**Table S1.** Evidence summary on Chinese medicine monomers for DR.

| **Therapeutic Strategy** | **Pathway**  **/Target** | **Typical Monomers** | **Administration Route** | **Collective Evidence and Potential** | **Translational** **Outlook** |
| --- | --- | --- | --- | --- | --- |
| Anti-angiogenesis | VEGF Signaling,  Hypoxic Response | Tanshinone IIA [7, 8],  Scutellaria barbata Polysaccharides [9],  Paeoniflorin [10],  Chlorogenic Acid [11] | IP,  Oral,  In vitro | Target the VEGF pathway upstream (HIF-1α) and downstream (MEK/ERK, ICAM-1), offering a broader anti-angiogenic approach that may overcome limitations of mono-target anti-VEGF therapies. | High potential as adjuncts to existing anti-VEGF biologics.  ^†^ Development of local, sustained-release ocular formulations is critical. |
| Neuroinflammation and Pyroptosis Inhibition | NLRP3,  NF-κB | Ginsenoside Rg1 [12], Puerarin [13, 14],  Dendrobium  Polysaccharides [15] | IP,  Oral | Dual protection: inhibiting neuronal apoptosis (via Bcl-2/Bax and Caspase-9) and suppressing neuroinflammation (via NLRP3 and NF-κB). | Potential for early intervention and combination therapy with vascular-targeting agents.  ^†^ Unique value in neurodegeneration. |
| Metabolic Regulation and Redox Homeostasis | AMPK/SIRT1,  NAD+ Metabolism | Ginsenoside Rd [16],  Notoginsenoside R1 [17],  Quercetin [18],  Curcumin [19] | IP,  Oral,  Unspecified | Converge on core metabolic sensors (AMPK, SIRT1) and antioxidant defense (Nrf2), improving NAD+ metabolism, mitochondrial function, and the intracellular redox state to counter fundamental DR drivers. | Targeting early metabolic dysfunction.  ^†^ Overcoming poor bioavailability is paramount for clinical success. |
| Novel Cell Death Intervention | Nrf2/GPX4 (Ferroptosis) | Resveratrol [20],  Curcumin [19],  Astragalus polysaccharides [21] | IP,  Oral | Represent a pioneering strategy by protecting Müller cells against ferroptosis via the Nrf2/GPX4 pathway. | Innovative potential for early disease halting. |
| Multi-Pathway and Network Modulation | Pathway Crosstalk,  miRNA Regulation | Ginsenoside Rb1/Re [22, 23],  Astragalus Polysaccharides [21] | IP,  Oral | Exhibit systems-level effects, modulating oxidative stress (SOD, CAT) and DNA damage (PARP) pathways, as well as miRNA networks, to influence SIRT1/ Nrf2/Bcl-2 pathways. | Their pleiotropic nature is advantageous for complex diseases. ^†^ Requiring precise standardization and targeted delivery systems. |

*Abbreviations:* VEGF: vascular endothelial growth factor, IP: intraperitoneal, HIF-1α: hypoxia-inducible factor-1 alpha, MEK: mitogen-activated protein kinase kinase, ERK: extracellular signal-regulated kinase, ICAM-1: intercellular adhesion molecule 1, NLRP3: nucleotide-binding domain, leucine-rich repeat-containing family, pyrin domain-containing 3, NF-κB: nuclear factor kappa-B, Bcl-2: B-cell lymphoma-2, Bax: Bcl-2-associated X protein, AMPK: AMP-activated protein kinase, SIRT1: sirtuin 1, NAD+: nicotinamide adenine dinucleotide, Nrf2: nuclear factor erythroid-derived 2-like 2, DR: diabetic retinopathy, GPX4: [glutathione peroxidase 4,](http://www.baidu.com/link?url=ub_PS_2btfREPqTGxka5BAdpPHbLinVYnoxZLsgGSc9B5ny_9_gtY8j_TKEWWlF_Z9ZFn6UrDvgVfpUG1GqiatgpV-q6YR5DS0BbR26k_UANnB6I5J4WKgkUMY2j4WAj) SOD: superoxide dismutase, CAT: catalase, PARP: poly (ADP-ribose) polymerase. ^†^ deserves special attention.

**Figures**


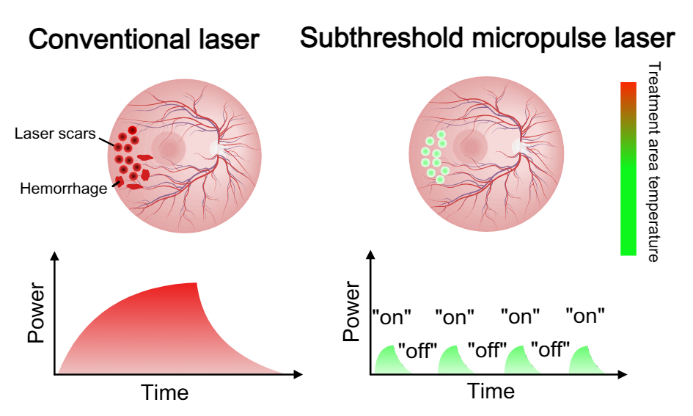


**Figure S1.** Emission mode of conventional laser and SML.

**References**

1. Abdelwahab, S.I., M.M.E. Taha, A. Farasani, et al., "Bibliometric analysis: A few suggestions (Part Two)," Curr Probl Cardiol 50,no.3 (2025): 102982.

2. Trujillo, C.M. and T.M. Long, "Document co-citation analysis to enhance transdisciplinary research," Sci Adv 4,no.1 (2018): e1701130.

3. van Eck, N.J. and L. Waltman, "Software survey: VOSviewer, a computer program for bibliometric mapping," Scientometrics 84,no.2 (2010): 523-538.

4. Tomaszewski, R., "Visibility, impact, and applications of bibliometric software tools through citation analysis," Scientometrics 128,no.7 (2023): 4007-4028.

5. Wells, J.A., A.R. Glassman, A.R. Ayala, et al., "Aflibercept, bevacizumab, or ranibizumab for diabetic macular edema," N Engl J Med 372,no.13 (2015): 1193-203.

6. Nguyen, Q.D., D.M. Brown, D.M. Marcus, et al., "Ranibizumab for diabetic macular edema: results from 2 phase III randomized trials: RISE and RIDE," Ophthalmology 119,no.4 (2012): 789-801.

7. Fan, K., S. Li, G. Liu, et al., "Tanshinone IIA inhibits high glucose‑induced proliferation, migration and vascularization of human retinal endothelial cells," Mol Med Rep 16,no.6 (2017): 9023-9028.

8. Qian, S., Y. Qian, D. Huo, et al., "Tanshinone IIa protects retinal endothelial cells against mitochondrial fission induced by methylglyoxal through glyoxalase 1," Eur J Pharmacol 857,no(2019): 172419.

9. Li, W. and H. Xiao, "Scutellaria barbata D. Don Polysaccharides Inhibit High Glucose-Induced Proliferation and Angiogenesis of Retinal Vascular Endothelial Cells," Diabetes Metab Syndr Obes 14,no(2021): 2431-2440.

10. Sun, W., R. Wang, K. Gong, et al., "Paeoniflorin-mediated downregulation of VEGFA: unveiling the therapeutic mechanism of buyang huanwu decoction in diabetic retinopathy," Naunyn Schmiedebergs Arch Pharmacol (2024).

11. Mei, X., L. Zhou, T. Zhang, et al., "Chlorogenic acid attenuates diabetic retinopathy by reducing VEGF expression and inhibiting VEGF-mediated retinal neoangiogenesis," Vascul Pharmacol 101,no(2018): 29-37.

12. Gao, Y., Y. Ji, Y. Luo, et al., "Ginsenoside Rg1 prevents early diabetic retinopathy via reducing retinal ganglion cell layer and inner nuclear layer cell apoptosis in db/db mice," Ann Transl Med 8,no.5 (2020): 232.

13. Kang, N.R., B.J. Pyun, D.H. Jung, et al., "Pueraria lobata Extract Protects Hydrogen Peroxide-Induced Human Retinal Pigment Epithelial Cells Death and Membrane Permeability," Evid Based Complement Alternat Med 2019,no(2019): 5710289.

14. Zhu, X., M. Xie, K. Wang, et al., "The effect of puerarin against IL-1β-mediated leukostasis and apoptosis in retinal capillary endothelial cells (TR-iBRB2)," Mol Vis 20,no(2014): 1815-23.

15. Yu, Z., C. Gong, B. Lu, et al., "Dendrobium chrysotoxum Lindl. alleviates diabetic retinopathy by preventing retinal inflammation and tight junction protein decrease," J Diabetes Res 2015,no(2015): 518317.

16. Tang, K., W. Qin, R. Wei, et al., "Ginsenoside Rd ameliorates high glucose-induced retinal endothelial injury through AMPK-STRT1 interdependence," Pharmacol Res 179,no(2022): 106123.

17. Fan, C., Y. Qiao, and M. Tang, "Notoginsenoside R1 attenuates high glucose-induced endothelial damage in rat retinal capillary endothelial cells by modulating the intracellular redox state," Drug Des Devel Ther 11,no(2017): 3343-3354.

18. Saikia, L., S.A.A. Barbhuiya, K. Saikia, et al., "Therapeutic Potential of Quercetin in Diabetic Neuropathy and Retinopathy: Exploring Molecular Mechanisms," Curr Top Med Chem 24,no.27 (2024): 2351-2361.

19. Xie, T., X. Chen, W. Chen, et al., "Curcumin is a Potential Adjuvant to Alleviates Diabetic Retinal Injury via Reducing Oxidative Stress and Maintaining Nrf2 Pathway Homeostasis," Front Pharmacol 12,no(2021): 796565.

20. Wang, Y., S.Y. Song, Y. Song, et al., "Resveratrol Protects Müller Cells Against Ferroptosis in the Early Stage of Diabetic Retinopathy by Regulating the Nrf2/GPx4/PTGS2 Pathway," Mol Neurobiol (2024).

21. Peng, Q.H., P. Tong, L.M. Gu, et al., "Astragalus polysaccharide attenuates metabolic memory-triggered ER stress and apoptosis via regulation of miR-204/SIRT1 axis in retinal pigment epithelial cells," Biosci Rep 40,no.1 (2020).

22. Dong, C., P. Liu, H. Wang, et al., "Ginsenoside Rb1 attenuates diabetic retinopathy in streptozotocin-induced diabetic rats1," Acta Cir Bras 34,no.2 (2019): e201900201.

23. Xie, W., P. Zhou, M. Qu, et al., "Corrigendum: Ginsenoside Re attenuates high glucose-induced RF/6A injury via regulating PI3K/AKT inhibited HIF-1a/VEGF signaling pathway," Front Pharmacol 15,no(2024): 1451696.
